# Kinetochore-associated Stu2 promotes chromosome biorientation in vivo

**DOI:** 10.1371/journal.pgen.1008423

**Published:** 2019-10-04

**Authors:** Matthew P. Miller, Rena K. Evans, Alex Zelter, Elisabeth A. Geyer, Michael J. MacCoss, Luke M. Rice, Trisha N. Davis, Charles L. Asbury, Sue Biggins

**Affiliations:** 1 Howard Hughes Medical Institute, Division of Basic Sciences, Fred Hutchinson Cancer Research Center, Seattle, Washington, United States of America; 2 Department of Biochemistry, University of Washington, Seattle, Washington 98195, United States of America; 3 Departments of Biophysics and Biochemistry, UT Southwestern Medical Center, Dallas, Texas, United States of America; 4 Department of Genome Sciences, University of Washington, Seattle, Washington, United States of America; 5 Department of Physiology & Biophysics, University of Washington, Seattle, Washington, United States of America; The University of North Carolina at Chapel Hill, UNITED STATES

## Abstract

Accurate segregation of chromosomes to daughter cells is a critical aspect of cell division. It requires the kinetochores on duplicated chromosomes to biorient, attaching to microtubules from opposite poles of the cell. Bioriented attachments come under tension, while incorrect attachments lack tension and must be released to allow proper attachments to form. A well-studied error correction pathway is mediated by the Aurora B kinase, which destabilizes low tension-bearing attachments. We recently discovered that in vitro, kinetochores display an additional intrinsic tension-sensing pathway that utilizes Stu2. The contribution of kinetochore-associated Stu2 to error correction in cells, however, was unknown. Here, we identify a Stu2 mutant that abolishes its kinetochore function and show that it causes biorientation defects in vivo. We also show that this Stu2-mediated pathway functions together with the Aurora B-mediated pathway. Altogether, our work indicates that cells employ multiple pathways to ensure biorientation and the accuracy of chromosome segregation.

## Author summary

The precise regulation of cell division is critical to processes such as self-renewal, proliferation and development. A key event in the cell cycle is the partitioning of every pair of duplicated chromosomes to daughter cells. Defects in chromosome partitioning lead to aneuploidy, a condition that is a common hallmark of cancer cells and the cause of some birth defects. Chromosomes segregate using their kinetochores, the specialized protein structures that are assembled on centromeric DNA sequences and attach to spindle microtubules. Here, we report that a protein that associates with kinetochores called Stu2 ensures that each kinetochore attaches to the proper microtubules. We identified a Stu2 mutant that does not associate with kinetochores and found that it generates aneuploidy. Together, our work identifies a previously unknown mechanism where cells ensure that chromosomes are accurately inherited during cell division.

## Introduction

Faithful partitioning of the genetic material is a fundamental aspect of cell division. Chromosome segregation errors are the most prevalent genetic alteration in tumor cells and are proposed to be a major factor in the evolution of cancer (reviewed in [1]). Segregation is mediated by the kinetochore, a highly conserved protein complex that physically attaches chromosomes to the spindle microtubules that ultimately pull the chromosomes apart in anaphase. To ensure that the duplicated chromosomes become segregated to each daughter cell, sister kinetochores must attach to microtubules from opposite poles, a state known as biorientation. Once kinetochores biorient, they come under tension from opposing microtubule-pulling forces. For this process to operate faithfully, kinetochores must “sense” whether proper bioriented microtubule attachments have been made and erroneous attachments must be corrected (reviewed in [2]). Pioneering work has yielded insight into the underlying mechanisms by showing that incorrect kinetochore attachments are unstable due to the absence of tension [3]. The selective release of attachments lacking tension gives the cell another chance to establish proper attachments.

The canonical “error correction” pathway is mediated by the conserved protein kinase Aurora B. The activity of Aurora B destabilizes kinetochore-microtubule interactions that are not under tension by phosphorylating multiple outer kinetochore components [4–8]. However, it is unclear whether this Aurora B-mediated pathway is solely responsible for correcting erroneous attachments. Recently, we discovered another pathway implicated in the correction of erroneous kinetochore-microtubule attachments in vitro [9,10]. Using a reconstitution system, we found (i) that kinetochores exhibit an intrinsic selectivity for high tension-bearing attachments that is independent of Aurora B [9], and (ii) that this direct mechano-sensitivity depends on the kinetochore-associated activity of the XMAP215 family member, Stu2 [10]. However, the extent to which kinetochore-associated Stu2 regulates kinetochore-microtubule attachments in vivo, as well as how it integrates with the Aurora B pathway, has yet to be determined.

Here, we address whether the kinetochore-associated function of Stu2 promotes biorientation in cells. Stu2 has multiple functions and regulates spindle microtubules (reviewed in [11]), so we looked for Stu2 mutations that abolish its kinetochore localization but that maintain normal length spindles. We found that a Stu2 mutant lacking its coiled-coil homodimerization domain fails to associate with kinetochores or to enhance kinetochore attachment stability in vitro. Expression of this mutant in cells results in defective kinetochore biorientation and leads to a spindle checkpoint-dependent cell cycle delay. Furthermore, we show that Stu2’s kinetochore function is necessary for the establishment of bioriented attachments but is dispensable thereafter, reminiscent of the requirement of Aurora B in error correction. Finally, kinetochore-associated Stu2 acts in concert with Aurora B; perturbing both pathways results in an additive growth defect and an increased rate of chromosome mis-segregation. Together, our findings suggest that mitotic error correction in vivo requires both the Aurora B-mediated and Stu2-dependent pathways. Our results provide further mechanistic insight into the process of error correction as well as the manner in which tension promotes accurate chromosome segregation.

## Results

### Dimerization-deficient mutant of Stu2 is defective in kinetochore association

**Fig 1:**
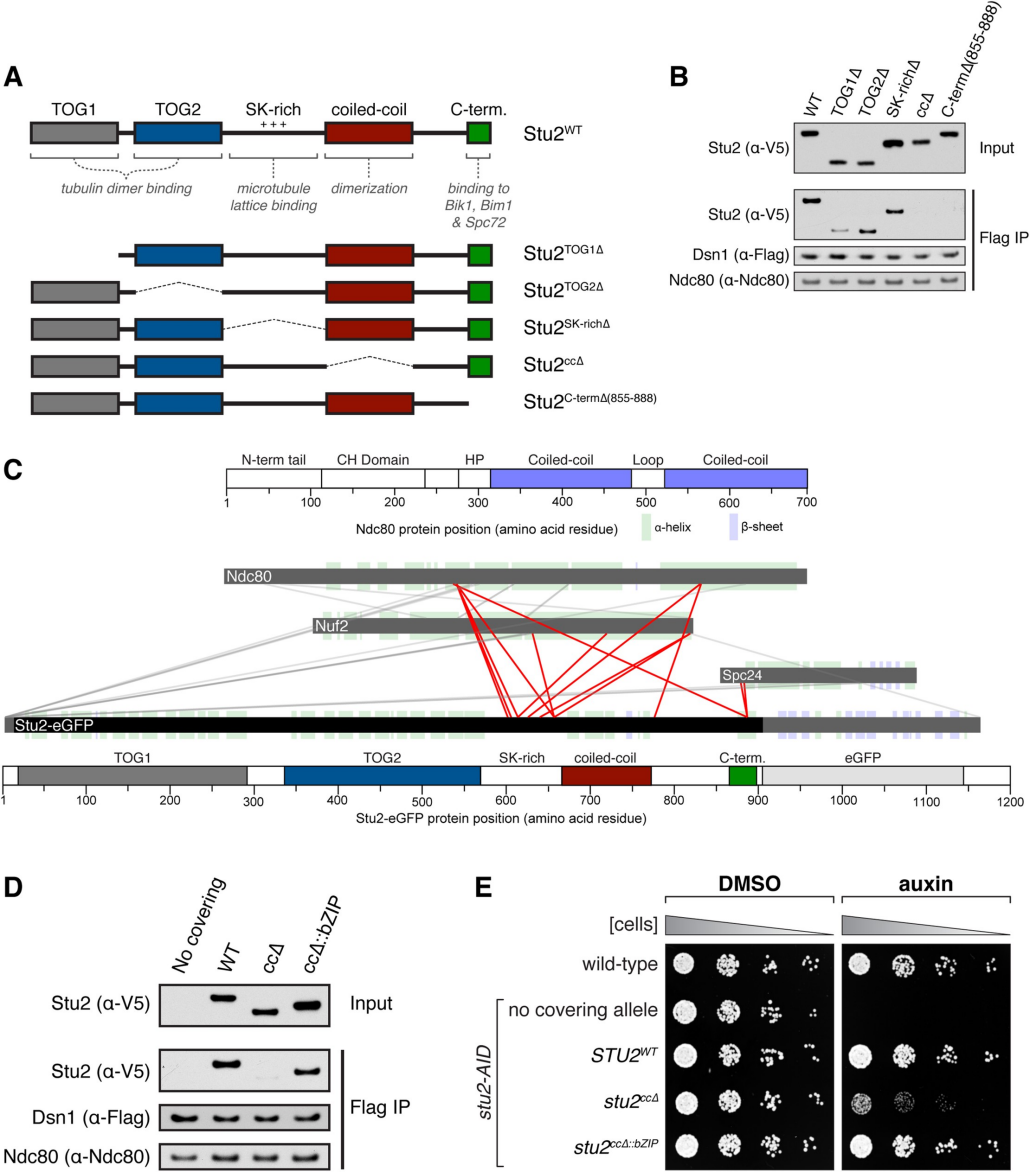
Dimerization of Stu2 is required for kinetochore association. A) Schematic of Stu2’s domain architecture and corresponding deletions. Note: Internal deletions (i.e. not at the N- or C-terminus) were made by inserting a linker, denoted by a dashed line. B) Exponentially growing *stu2-AID* cultures expressing an ectopic copy of *STU2* (*STU2*^*WT*^, SBY13901; *stu2*^*TOG1Δ*^, SBY13904; *stu2*^*TOG2Δ*^, SBY13907; *stu2*^*SK-richΔ*^, SBY13913; *stu2*^*ccΔ*^, SBY13916; or *stu2*^*C-termΔ(855–888)*^, SBY14269) that also contained Dsn1-6His-3Flag were treated with auxin 30 min prior to harvesting. Protein lysates were subsequently prepared and kinetochore particles were purified by α-Flag immunoprecipitation (IP) and analyzed by immunoblotting. C) Cross-linking mass spectrometry analysis reveals interactions between recombinant Stu2-eGFP and the Ndc80 complex. Cross-links formed with EDC between Stu2 and the Ndc80 complex are depicted by red lines. Bar diagrams with structural features of Ndc80 and Stu2-eGFP proteins are included. (CH: calponin homology; HP: hairpin; TOG: tumor overexpressed gene; SK-rich: regions with stretches of sequences rich in Serine, Glycine, Lysine). For clarity, cross-links within Ndc80 complex proteins and those involving Stu2 fusion regions (extreme N-terminus and eGFP) are shown in grey. Data are shown for peptides with Percolator assigned q-values ≤ 0.05 and a minimum of 2 PSMs. D) Exponentially growing Stu2-AID cultures with an ectopic copy of *STU2* (*STU2*^*WT*^, SBY13901; *stu2*^*ccΔ*^, SBY13916; or *stu2*^*ccΔ*::*bZIP*^, SBY13935) or without an ectopic allele (no covering allele, SBY13772) that also contained Dsn1-6His-3Flag were treated with auxin 30 min prior to harvesting. Protein lysates were subsequently prepared and kinetochore particles were purified by α-Flag immunoprecipitation (IP) and analyzed by immunoblotting. E) Wild-type (SBY3), *stu2-AID* (no covering allele, SBY13772) and *stu2-AID* cells expressing various *STU2-3V5* alleles from an ectopic locus (*STU2*^*WT*^, SBY13901; *stu2*^*ccΔ*^, SBY13916; *stu2*^*ccΔ*::*bZIP*^, SBY13935) were serially diluted (5-fold) and spotted on plates containing either DMSO or 500 μM auxin. Refer to S2 Fig for a similar analysis of all alleles examined.

Stu2 has important roles in both the formation of the mitotic spindle and the regulation of kinetochore function [10,12–14]. With the goal of identifying a mutant that disrupts Stu2’s kinetochore localization but supports normal mitotic spindle formation, we set out to 1) map the domain(s) required for its kinetochore association, and 2) examine the spindle length in cells expressing these mutants. XMAP215 family members are large proteins that contain variable numbers of highly conserved tumor over-expressed gene (TOG) domain arrays that bind to curved tubulin dimers [12,15–17] as well as a basic linker domain that promotes binding to the microtubule lattice [18,19]. The budding yeast Stu2 protein contains two N-terminal TOG domains, followed by a basic linker domain (SK-rich), a homo-dimerization domain (coiled-coil), and a small C-terminal domain that is required for Stu2’s association with the microtubule-associated proteins (MAPs) Bik1, Bim1 and Spc72 (Fig 1A; [12,19–21]).

To determine the element(s) within Stu2 required for kinetochore-association, we individually deleted each of these domains and expressed the mutant alleles from an ectopic locus in a strain harboring a conditional *stu2-AID* allele at the endogenous locus [10]. Each of these mutant alleles was expressed at or near wild-type levels (Fig 1B & S1E Fig, inputs). The localization of a similar set of *stu2* mutations was recently examined in cells [22], but only in the presence of wild-type endogenous protein, which through homo-dimerization and/or competition could affect the localization of the mutant proteins. We therefore sought to examine the mutants in the absence of endogenous protein. While we attempted to examine their kinetochore localization in cells using GFP-tagged alleles, it was not possible to distinguish the pool of Stu2 on the microtubule tip from that on the kinetochore. We therefore analyzed the levels of the Stu2 mutants on purified kinetochores by immunoblotting. We depleted the endogenous Stu2-AID protein by adding auxin and then isolated native kinetochores via single-step immunoprecipitation of the Mis12/MIND/Mtw1 complex component Dsn1-His-Flag [9]. While most of the mutations did not affect the Stu2-kinetochore interaction, deletion of either the coiled-coil or C-terminal domains disrupted Stu2’s ability to co-purify with native kinetochores, and deletion of the TOG1 domain dramatically reduced Stu2’s kinetochore interaction (Fig 1B & S1E Fig).

To further confirm the requirement of these domains, we performed in vitro binding assays. We purified and immobilized kinetochores lacking endogenous Stu2 and examined the ability of each Stu2 variant (purified independently) to bind the immobilized kinetochores. Consistent with our co-purification results, deletions of the coiled-coil, C-terminal, and TOG1 domains disrupted the ability of purified Stu2 to ‘re-bind’ the kinetochores (S1A–S1D Fig). These in vitro binding data are largely consistent with the localization data of similar mutants analyzed by microscopy in cells containing the endogenous protein [22]; a summary of in vitro binding data can be found in S1G Fig. However, our in vitro binding experiments are conducted in the absence of microtubules and allow us to distinguish direct kinetochore binding from microtubule tip association, unlike experiments in yeast cells where the kinetochores are constitutively associated with microtubules [23–25].

As a complementary approach to define regions of Stu2 required for its kinetochore interaction, we conducted crosslinking mass spectrometry with recombinant Stu2-eGFP and its outer kinetochore receptor, the Ndc80 complex (Ndc80c) (S1F Fig; [10,26,27]). This method provides information on the proximity of various residues to each other; thus, crosslinks are expected between protein-protein interfaces that directly mediate binding interactions as well as transient interaction nodes that perhaps play a regulatory role. Consistent with previous reports, we observed intra-complex crosslinks within the heterotetrameric Ndc80c [28,29]. We also found numerous crosslinks between Ndc80c and Stu2. We identified several crosslinks between the C-terminal domain of Stu2 and Ndc80c, consistent with our observation that the C-terminus is required for kinetochore association (Fig 1C). There were also many crosslinks between Stu2’s basic linker (SK-rich) domain and Ndc80c. This observation was unexpected since Stu2’s basic linker is completely dispensable for Stu2-Ndc80c binding (Fig 1B & S1F Fig), suggesting that Stu2’s basic linker interacts with Ndc80c in a transient, weak fashion rather than forming a strong, stable bond.

Interestingly, even though Stu2’s coiled-coil domain is required for its kinetochore association, we did not detect any crosslinks between this domain and Ndc80c. This observation suggests that Stu2’s coiled-coil domain may not contain sequence-specific elements that directly mediate Ndc80c binding; instead, the protein may simply need to form a homo-dimer. Consistent with this, replacement of the coiled-coil domain with the homo-dimerization of the Gcn4 bZIP transcriptional activator [30] restored Stu2‘s kinetochore association (Fig 1D; [22]).

We next examined the effect of disrupting the Stu2-kinetochore interaction on cell growth. Cells expressing the coiled-coil domain mutant (henceforth referred to as *stu2*^*ccΔ*^) exhibited impaired growth and benomyl sensitivity when the endogenous Stu2-AID protein was depleted (Fig 1E and S2 Fig; [10,12,14,16]). Restoring the ability of Stu2 to homo-dimerize and to associate stably with kinetochores using the *stu2*^*ccΔ*::*bZIP*^ allele also fully rescued the growth defects of the coiled-coil deletion. These observations strongly suggest that kinetochore-localized Stu2 is important for normal cell growth.

### Dimerization-deficient *stu2* mutant supports normal mitotic spindle formation in cells

**Fig 2:**
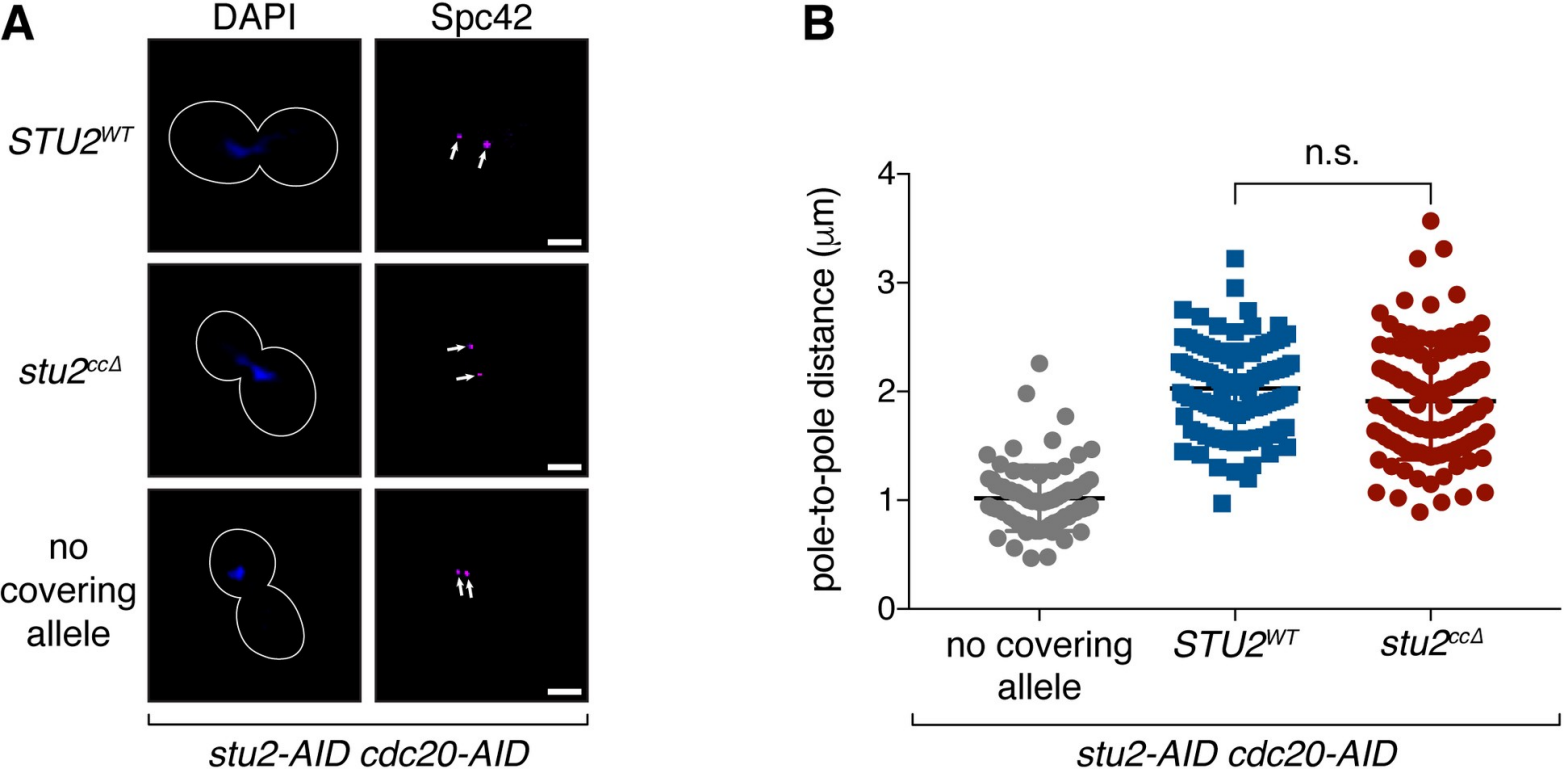
Dimerization-deficient mutant of Stu2 supports normal mitotic spindle formation. A) Exponentially growing *stu2-AID cdc20-AID* cultures with an ectopically expressed *STU2* allele (*STU2*^*WT*^, SBY17369; *stu2*^*ccΔ*^, SBY17371) or without an ectopic allele (no covering allele, SBY17367) that also contained *MTW1-3GFP* (kinetochore) and *SPC42-CFP* (spindle pole; marked with white arrows) were treated with auxin for 2 h to arrest cells in metaphase. Representative images for each are shown. White bars represent 2 μm. B)Spindle length (spindle pole-to-pole distance) and kinetochore distribution (distance between bi-lobed kinetochore clusters; Fig 4C) was measured for cells described in (A). n = 80–105 cells; p values were determined using a two-tailed unpaired t test (n.s. = not significant).

We sought a Stu2 mutant that lacked kinetochore association but maintained spindle and microtubule functions [12–14]. While the coiled-coil, TOG1, and C-terminal domains are required for Stu2's kinetochore association, the TOG1 and C-terminal domains have established roles in tubulin binding [15,18,31] and/or interactions with proteins involved in microtubule function [20,21,32], respectively. Because the coiled-coil domain is not known to interact with tubulin or other proteins, we analyzed spindle length in the *stu2*^*ccΔ*^ mutant by arresting cells in metaphase (by depleting Cdc20 using a *cdc20-AID* allele) and measuring the distance between spindle poles (labeled by Spc42-CFP). We performed this experiment in cells carrying the *stu2-AID* allele with an ectopic copy of the *STU2* variant to be examined. As expected, *stu2-AID* cells that were treated with auxin, and that did not express a covering *STU2* allele, arrested with a short bipolar spindle (1.02 ± 0.30 μm distance between Spc42 foci; [10,13,14]), whereas cells that expressed a wild-type copy of *STU2* arrested with a longer and characteristic metaphase-length spindle (2.03 ± 0.40 μm; Fig 2A & 2B). Cells that expressed the *stu2*^*ccΔ*^ allele arrested with a normal spindle length (1.91 ± 0.54 μm). Thus, while mutants that alter Stu2’s dimerization exhibit polymerization defects in vitro [12,18], this does not lead to a defect in spindle formation in vivo. Because the *stu2*^*ccΔ*^ mutant is defective in kinetochore binding yet supports a normal mitotic spindle, it provides a tool to specifically examine Stu2’s kinetochore function.

### Kinetochore association is required for Stu2’s ability to enhance attachment stability in vitro

**Fig 3:**
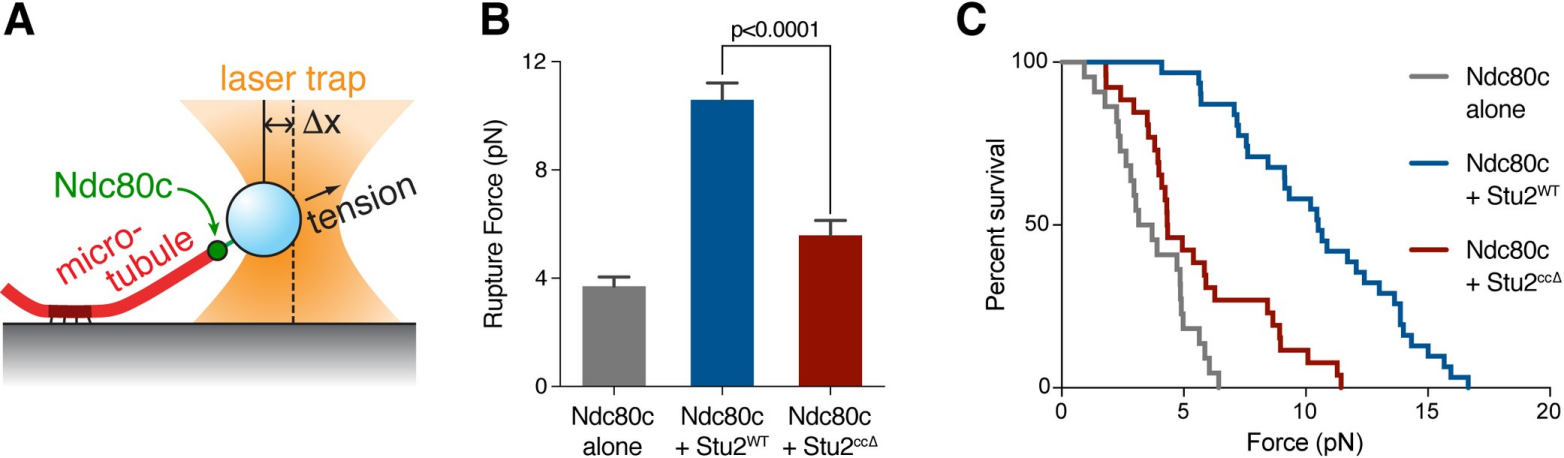
Kinetochore-binding by Stu2 is required for enhancing Ndc80c-microtubule attachment stability. A) Schematic of optical trap assay. Dynamic microtubules are grown from coverslip-anchored seeds. Beads linked to Ndc80c via Spc24 are manipulated using an optical trap to exert applied force across the Ndc80c-microtubule interface in the presence or absence of purified Stu2. B) Mean rupture forces for Ndc80c-linked beads untreated or incubated with free Stu2^WT^ or Stu2^ccΔ^. Error bars represent SEM (n = 22–31 events). P value was determined using a two-tailed unpaired t test. All measurements were conducted within the same experimental set, but the data for Ndc80c alone and in the presence of Stu2^WT^ are reported previously in [10]. C) Attachment survival probability versus force for the data in (B).

We previously found that Stu2 significantly strengthened Ndc80-based attachments in vitro [10], so we next tested whether the Stu2^cc*Δ*^ mutant disrupted this function. As before, we used an optical trapping-based ‘‘force-ramp” technique in which native Ndc80c was linked to beads and then attached to growing microtubule tips using a laser trap (Fig 3A; reviewed in [33]). We gradually increased the force across the Ndc80c-microtubule interface until the attachment ruptured. When beads contained Ndc80c alone, they formed attachments with an average rupture strength of 3.7 ± 0.3 pN [10,34]. Addition of purified wild-type Stu2 increased the rupture strength of these Ndc80c-based attachments dramatically, to an average of 10.6 ± 0.6 pN [10]. In contrast, the strength of Ndc80-based attachments was much lower upon addition of the purified Stu2^ccΔ^ mutant, with an average rupture strength of only 5.6 ± 0.6 pN (Fig 3B & 3C). The small enhancement of attachment strength observed for the Stu2^ccΔ^ mutant is likely due to the small (but detectable) residual kinetochore/Ndc80c binding of this mutant (see Fig 1D). Overall, these data show that the Stu2^ccΔ^ mutant disrupts one of its kinetochore functions in vitro, and furthermore, is consistent with kinetochore-association of Stu2 being required for these activities.

### Perturbing Stu2 kinetochore association results in a kinetochore attachment defect and spindle checkpoint-dependent cell cycle delay

**Fig 4:**
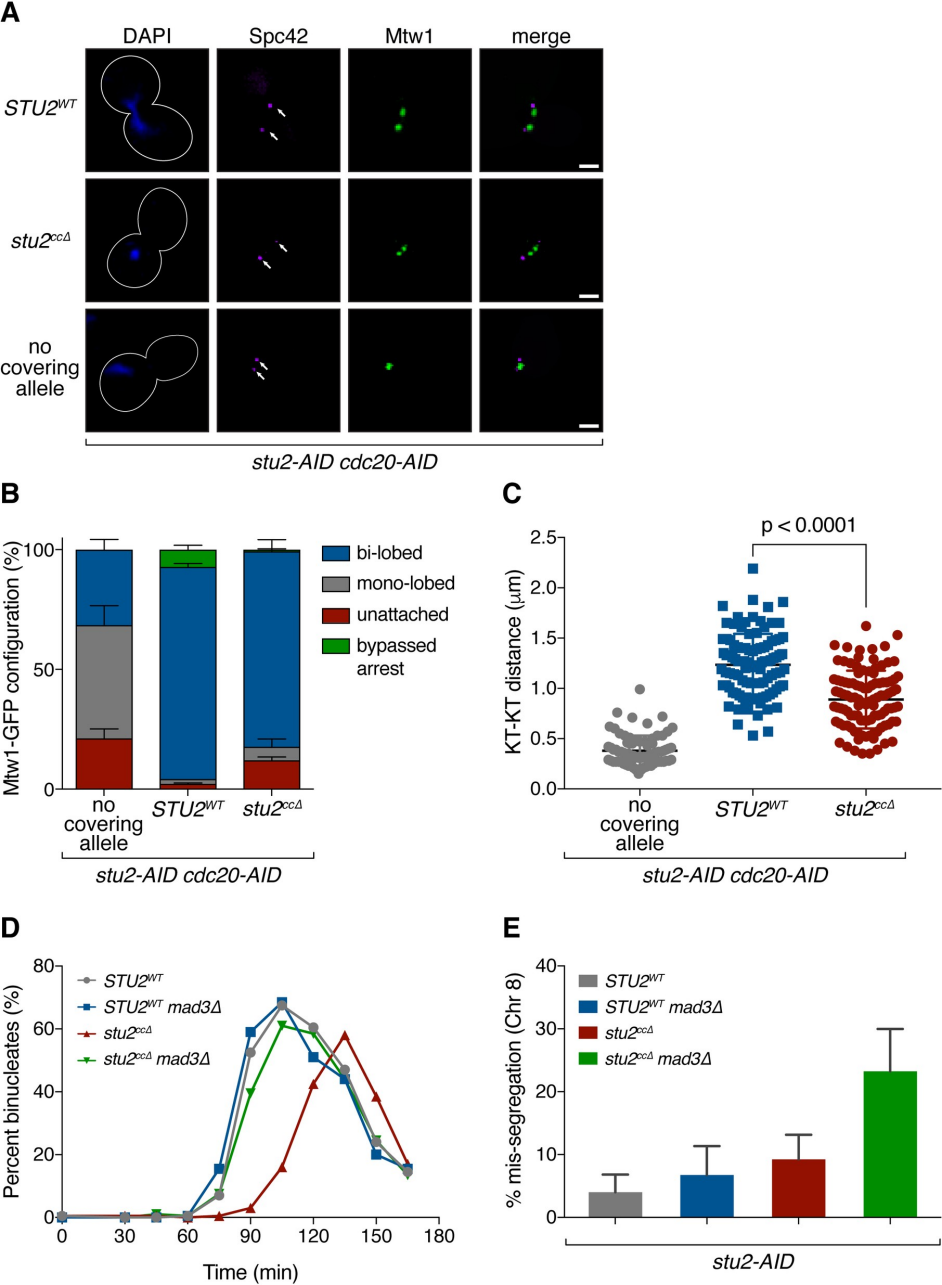
Kinetochore-binding deficient Stu2 exhibits a biorientation defect and spindle checkpoint-dependent cell cycle delay. A) Exponentially growing *stu2-AID cdc20-AID* cultures with an ectopically expressed *STU2* allele (*STU2*^*WT*^, SBY17369; *stu2*^*ccΔ*^, SBY17371) or without an ectopic allele (no covering allele, SBY17367) that also contained *MTW1-3GFP* (kinetochore) and *SPC42-CFP* (spindle pole; marked with white arrows) were treated with auxin for 2 h to arrest cells in metaphase. Representative images for each are shown. White bars represent 2 μm. B) Quantification of Mtw1 localization from (A). Error bars represent SD of two independent experiments; n = 100 cells for each experiment. C) Kinetochore distribution (distance between bi-lobed kinetochore clusters) and spindle length (spindle pole-to-pole distance; Fig 2B) was measured for cells described in (A). n = 80–105 cells; p values were determined using a two-tailed unpaired t test. D) *stu2-AID* cells with an ectopically expressed *STU2* allele with and without a spindle checkpoint mutation (*STU2*^*WT*^ *MAD3*, SBY17527; *stu2*^*ccΔ*^ *MAD3*, SBY17560; *STU2*^*WT*^ *mad3Δ*, SBY17668; *stu2*^*ccΔ*^ *mad3Δ*, SBY17669) that also contained a fluorescently labeled *CEN8* were released from a G1 arrest into auxin containing media. Cell cycle progression determined by the accumulation of binucleate cells. Shown is a representative experiment. E) Quantification of chromosome mis-segregation in anaphase (percent of binucleate cells with a fluorescently labeled *CEN8* signal in only one of the two nuclei) from (D). Error bars represent SD of two independent experiments; n = 200 cells for each experiment.

We next examined the effects of removing Stu2 from kinetochores in vivo. To do this, we first analyzed metaphase kinetochore distribution in *stu2^ccΔ^* mutant cells. In budding yeast, properly attached bioriented kinetochores cluster and exhibit a characteristic bi-lobed distribution at metaphase when they come under tension [23–25]. Cells were therefore arrested in metaphase by degrading Cdc20 (using a *cdc20-AID* allele) and kinetochore distribution was assayed using Mtw1-3GFP. As expected, the vast majority of *stu2-AID* cells treated with auxin that expressed a wild-type allele of *STU2* exhibited normal spindle length and a normal bi-lobed kinetochore distribution (89% ± 1%) (Fig 4A & 4B). Consistent with previous observations [10,13,14,35,36], cells depleted of Stu2 had extremely short spindles and three classes of kinetochore configurations: bi-lobed (32% ± 4%), mono-lobed (47% ± 8%), and unattached/off spindle axis (21% ± 4%; [37]). In contrast, the *stu2-AID* cells expressing the *stu2*^*ccΔ*^ allele had normal length spindles but a significantly altered kinetochore alignment (Fig 2A and 2B & Fig 4A and 4B). Although the majority of *stu2*^*ccΔ*^ cells displayed a bi-lobed kinetochore configuration (82% ± 5%), the clusters appeared closer together than *STU2*^*WT*^ and some cells also displayed unattached kinetochores (12% ± 1%; Fig 4B). We measured the distance between the Mtw1-3GFP foci in cells displaying a bi-lobed kinetochore configuration. The kinetochore foci were 1.23 ± 0.31 μm apart in cells expressing *STU2*^*WT*^ (Fig 4A & 4C). The cells expressing *stu2*^*ccΔ*^ exhibited a significantly shorter distance of 0.89 ± 0.29 μm between kinetochore foci (compared to the 0.38 ± 0.15 μm for cells not expressing a copy of *STU2*; Fig 4A & 4C). This shorter distance between kinetochore foci was consistent across all spindle lengths examined (S3A Fig), suggesting that the kinetochores do not come under normal levels of tension in the *stu2*^*ccΔ*^ cells. Together, these results suggest that abolishing Stu2's kinetochore localization in vivo results in the formation of spindles with normal length but leads to defects in kinetochore tension and attachments.

Defects in kinetochore-microtubule interactions trigger the spindle checkpoint and delay anaphase onset until they are corrected (reviewed in [38]). Thus, we examined whether the kinetochore alignment defects observed in the *stu2*^*ccΔ*^ expressing cells cause a cell cycle delay. Cells were arrested in G1 and released into auxin-containing media to degrade the endogenous Stu2-AID protein, and ectopically expressed either *STU2*^*WT*^ or *stu2*^*ccΔ*^. Anaphase was delayed in cells expressing *stu2*^*ccΔ*^ (Fig 4D & S3B–S3F Fig), suggesting spindle checkpoint activation. Deletion of the spindle checkpoint component *MAD3* suppressed the cell cycle delay and allowed *stu2*^*ccΔ*^ cells to progress into anaphase (Fig 4D), consistent with the delay being caused by incorrect kinetochore attachments.

We next analyzed the frequency of chromosome mis-segregation in *stu2*^*ccΔ*^ mutants when the spindle checkpoint was abolished using a fluorescently marked centromere on chromosome VIII. *stu2-AID* cells ectopically expressing either *STU2*^*WT*^ or *stu2*^*ccΔ*^ in the presence or absence of *MAD3* were released from a G1 arrest into auxin-containing media. The mis-segregation of chromosome VIII was notably increased in *stu2*^*ccΔ*^ *mad3Δ* cells (*STU2*^*WT*^ *MAD3*, 4.0 ± 2.8%; *STU2*^*WT*^ *mad3Δ*, 6.8 ± 4.6%; *stu2*^*ccΔ*^ *MAD3*, 9.3 ± 3.9%; *stu2*^*ccΔ*^ *mad3Δ*, 23.3 ± 4.8%; Fig 4E). Taken together, these data suggest that Stu2 localization to kinetochores is dispensable for the formation of a normal mitotic spindle, yet required for correct kinetochore attachments in vivo.

### Kinetochore-associated Stu2 is required for the establishment of bioriented attachments but dispensable for their maintenance

**Fig 5:**
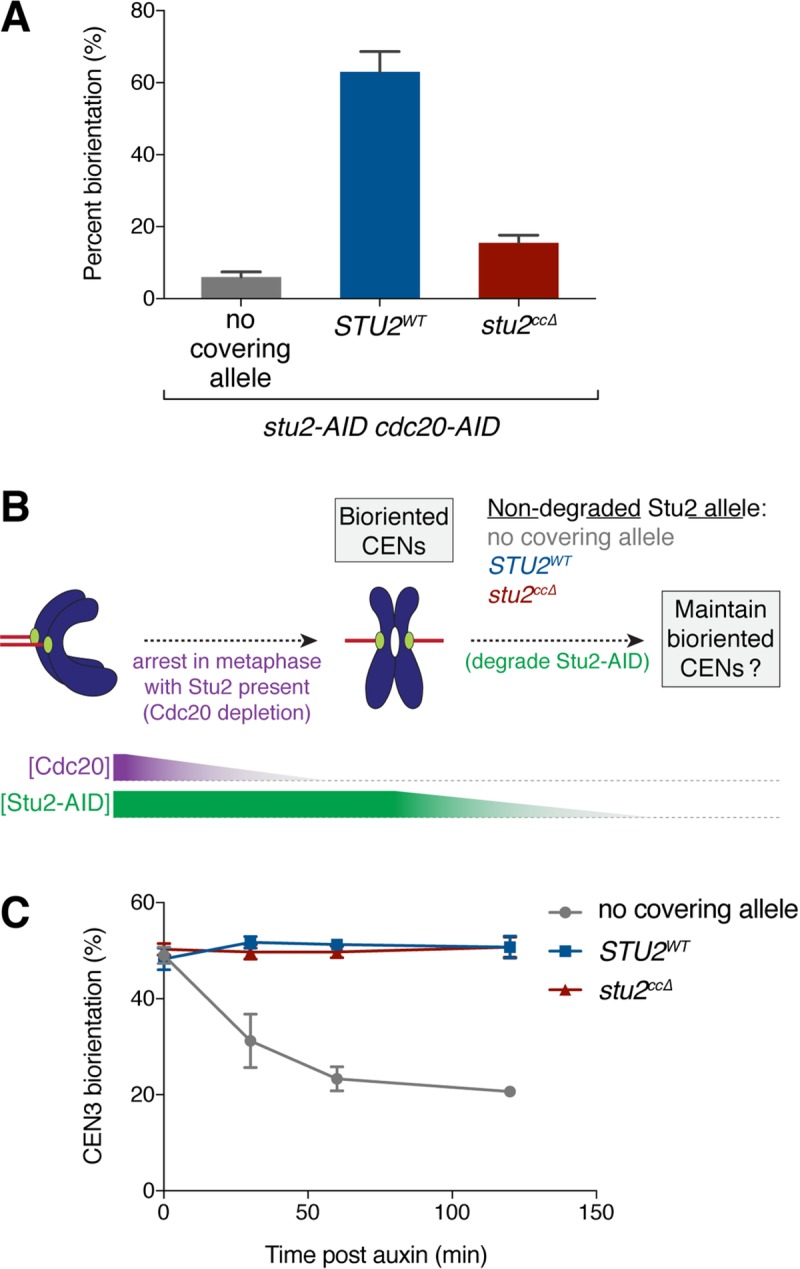
Kinetochore-associated Stu2 is not required for maintenance of biorientation. A) Exponentially growing *stu2-AID cdc20-AID* cultures with an ectopically expressed *STU2* allele (*STU2*^*WT*^, SBY17748; *stu2*^*ccΔ*^, SBY17750) or without an ectopic allele (no covering allele, SBY17708) that also contained a fluorescently labeled *CEN8* were treated with auxin for 2 h to arrest cells in metaphase. Percentage of cells that display two distinct GFP foci (i.e. bioriented *CEN8*) was quantified. Shown is the average of two independent experiments, error bars represent SD (n ≧ 100 cells). B) Schematic of experiment in (C). C) Exponentially growing *stu2-AID pMET-CDC20* cells that contained a fluorescently labeled *CEN3* and an ectopically expressed *STU2* allele (*STU2*^*WT*^, SBY18370; *stu2*^*ccΔ*^, SBY18371) or without an ectopic allele (no covering allele, SBY18359) were arrested in metaphase by the addition of methionine for 3 h. Once arrested in metaphase, auxin was added to degrade the Stu2-AID protein and the percentage of cells that display two distinct GFP foci (i.e. bioriented *CEN3*) was quantified over time. Error bars represent SD of three independent experiments; n = 100 cells for each time point.

Tension sensing is critical for the establishment of correctly bioriented attachments (reviewed in [2]). We previously found that Stu2 is required for the tension-dependent stabilization of microtubule attachments in vitro [10], potentially implicating this intrinsic tension sensing pathway in the process of biorientation. Our observation that cells expressing the kinetochore-binding deficient *stu2*^*ccΔ*^ allele display abnormal kinetochore clustering (Fig 4A–4C) is consistent with defects in the ability to correctly biorient and come under the proper level of tension. To directly examine whether there was a biorientation defect in *stu2*^*ccΔ*^ cells, we fluorescently marked the centromere of chromosome VIII [39] and arrested cells in metaphase to determine whether the sister kinetochores can biorient due to the opposing microtubule pulling forces [25]. In metaphase cells expressing a wild-type copy of *STU2*, 63.0± 6% displayed bioriented attachments, as judged by the fluorescently marked centromeres appearing as two distinct foci (Fig 5A). As expected, cells that did not express a covering allele of *STU2* had only 6.0 ± 1% of cells with bioriented attachments. Cells expressing the *stu2*^*ccΔ*^ allele also displayed a severe biorientation defect, with only 15.5 ± 2% of cells displaying two distinct centromere-marked foci.

While our data are consistent with the *stu2*^*ccΔ*^ allele specifically disrupting the function of kinetochore-associated Stu2, it was recently reported that a similar Stu2 mutant protein that lacks the coiled-coil as well as its C-terminal domain displays reduced microtubule polymerase activity in vitro [18]. In addition, previous studies showed that Stu2’s in vitro microtubule binding is reduced when dimerization is prevented [12]. To rule out the possibility that the observed biorientation defects are due to decreased microtubule polymerase activity, we examined the phenotypes of cells expressing a *stu2* mutant that partially restored microtubule polymerase activity in vitro. This mutant adds positively charged residues to the basic linker domain to restore the dimer-equivalent number of basic residues [18]. This mutant phenocopied the *stu2*^*ccΔ*^ mutant, displaying equivalent defects in both kinetochore association as well as biorientation (S4A–S4C Fig), suggesting that the observed biorientation defects do not correlate with the amount of microtubule polymerase activity. Together, these findings indicate that kinetochore-associated Stu2 plays an important role in the *establishment* of bioriented attachments in vivo, similar to the Aurora B-mediated error correction pathway.

Aurora B function is dispensable once bioriented attachments have been made [8], so we tested whether this was also the case for kinetochore-associated Stu2. To assay the maintenance of biorientation, we arrested *stu2-AID* cells containing a fluorescently marked *CEN3* in metaphase (using a methionine repressible *pMET-CDC20* allele) to establish biorientation (Fig 5B). We then added auxin to deplete the endogenous Stu2-AID protein and analyzed the maintenance of *CEN3* biorientation. As expected, cells expressing *STU2*^*WT*^ maintained bioriented attachments after the addition of auxin (50.7 ± 2.3% at 2 h post auxin addition; Fig 5C). In cells without a covering *STU2* allele, however, the number of cells with bioriented attachments quickly decreased after the addition of auxin (20.7 ± 0.6% at 2 h post auxin), likely due to mitotic spindle collapse (S4D–S4F Fig). In contrast, in cells expressing the *stu2*^*ccΔ*^ allele, the level of biorientation was maintained after the addition of auxin and appeared indistinguishable from that of wild-type (50.7 ± 2.1%). These results suggest that Stu2’s kinetochore-associated function is only required for the *establishment* of correctly bioriented attachments and is dispensable thereafter.

### Kinetochore association-deficient mutant of Stu2 displays a synthetic phenotype with an Aurora B mutant

**Fig 6:**
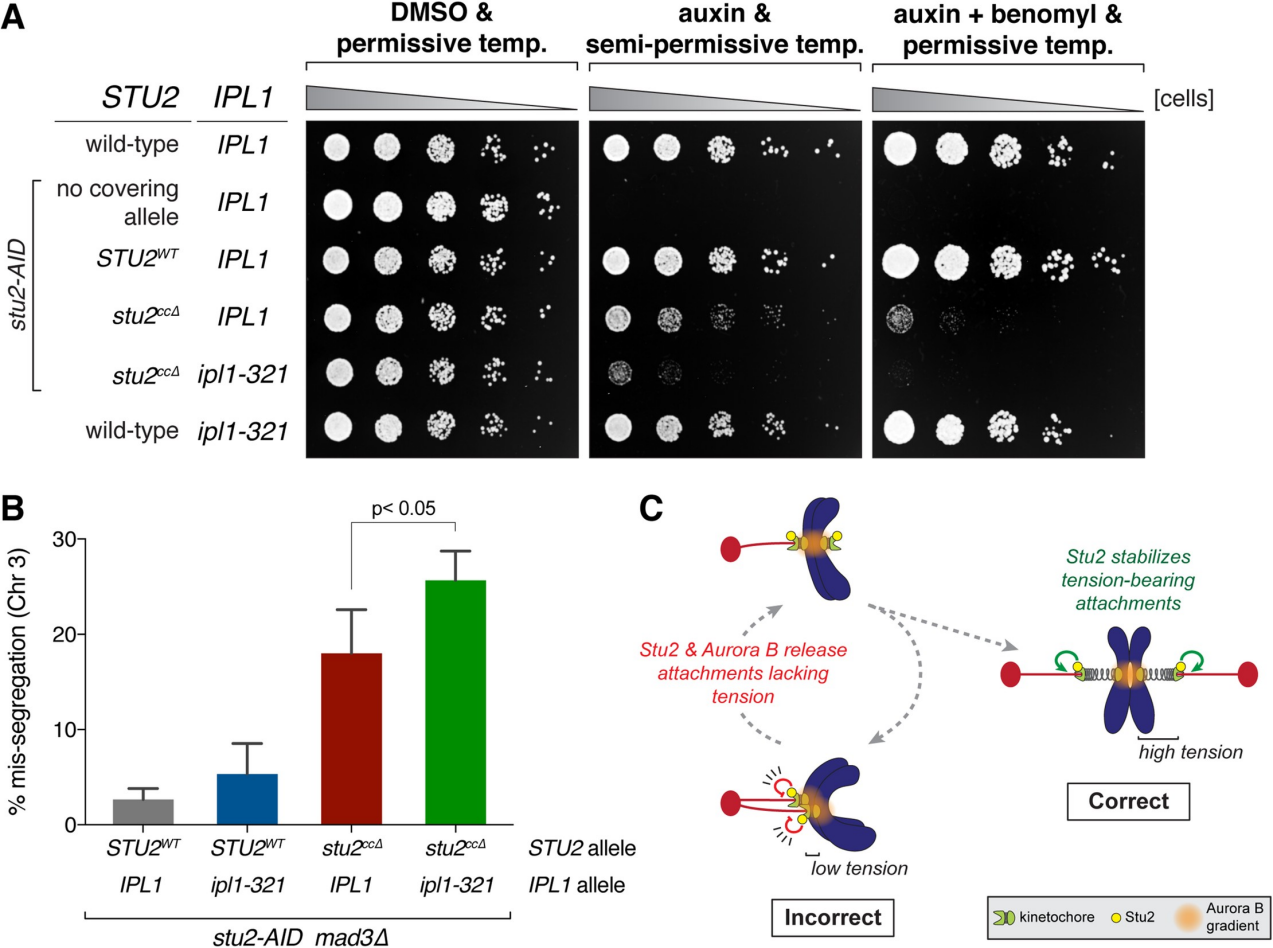
Mutants in Stu2 and Aurora B show a synergistic biorientation defect. A) Wild-type (SBY3), *stu2-AID* (no covering allele, SBY13772) and *stu2-AID* cells expressing various *STU2-3V5* alleles from an ectopic locus (*STU2*^*WT*^, SBY13903; *stu2*^*ccΔ*^, SBY13918) or also containing an *ipl1-321* allele (*stu2*^*ccΔ*^ *ipl1-321*, SBY17100) or *ipl1-321* alone (SBY630) were serially diluted (5-fold) and spotted on YPD or 5 μg/ml benomyl plates containing either DMSO or 500 μM auxin and incubated at 23°C (permissive) or 30°C (semi-permissive). B) Exponentially growing *stu2-AID mad3Δ* cells containing or lacking an *ipl1-321* allele and also an ectopically expressed *STU2* allele (*STU2*^*WT*^ *IPL1*, SBY18242; *STU2*^*WT*^ *ipl1-321*, SBY18244; *stu2*^*ccΔ*^ *IPL1*, SBY18246; *stu2*^*ccΔ*^ *ipl1-321*, SBY18248) that also contained a fluorescently labeled *CEN3* were arrested in G1 at the permissive temperature (23°C) and subsequently released from the G1 arrest into auxin containing media at a semi-permissive temperature (30°C). Quantification of chromosome mis-segregation in anaphase is shown. Error bars represent SD of three independent experiments; n = 100 cells for each experiment. p value was determined using a two-tailed paired t test. C) Model: Kinetochore-associated Stu2 confers tension sensitivity to kinetochore-microtubule attachments and is required for the establishment of bioriented attachments in vivo. Stu2 and Aurora B function together to release (destabilize) low tension bearing/incorrect kinetochore-microtubule attachments in cells. At high tension, a second function of Stu2 serves to stabilize correct attachments.

Our work suggests that kinetochore bound Stu2 promotes biorientation, so we tested its contribution relative to the Aurora B-mediated error correction pathway. First, we examined whether perturbing Stu2 kinetochore association enhanced the defects of an Aurora B mutant using the *stu2*^*ccΔ*^ mutant and the *ipl1-321* temperature sensitive allele of Aurora B [4,40]. Since Aurora B activity is essential for cell viability, we used a semi-permissive temperature in which *ipl1-321* cells grow nearly as well as wild-type cells. We found that under conditions where both pathways were perturbed (i.e. in the presence of auxin and at the semi-permissive temperature of 30°C), the cells expressing the *stu2*^*ccΔ*^ allele and harboring *ipl1-321* displayed a significant additive growth defect over either of the single mutations (Fig 6A & S5 Fig). We also observed a similar additive growth defect for the double mutant when grown at the permissive temperature (23°C) but in the presence of low concentrations of benomyl (Fig 6A).

Finally, we asked whether the additive growth defects are correlated with an increase in chromosome mis-segregation. As above, we used the *stu2*^*ccΔ*^ mutant and the *ipl1-321* allele and cells containing a fluorescently marked centromere of chromosome III to monitor chromosome segregation. We released cells from a G1 arrest into auxin containing media (to degrade the endogenous Stu2-AID protein) and at the semi-permissive temperature of 30°C and examined chromosome mis-segregation in anaphase (i.e. the percentage of cells that failed to segregate a copy of chromosome III to each of the resulting daughter nuclei). As expected, control cells expressing wild-type *STU2* and *IPL1*, as well as *ipl1-321* cells with the Aurora B pathway partially disrupted alone, displayed low levels of chromosome mis-segregation (*STU2*^*WT*^ *IPL1*, 2.7 ± 1.2%; *STU2*^*WT*^ *ipl1-321*, 5.3 ± 3.2%; Fig 6B). Cells expressing the kinetochore association-deficient *stu2*^*ccΔ*^ mutant alone showed an enhanced level of chromosome mis-segregation (*stu2*^*ccΔ*^ *IPL1*, 18.0 ± 4.6%), however, the *stu2*^*ccΔ*^ *ipl1-321* double mutant cells displayed a significant increase in the rate of chromosome mis-segregation compared to either single mutant alone (*stu2*^*ccΔ*^ *ipl1-321*, 25.7 ± 3.1%; Fig 6B). Altogether, these results suggest that the kinetochore bound pool of Stu2 plays an important role in establishing correct bioriented attachments in vivo, and that this mechanism appears to work in concert with the canonical Aurora B-mediated error correction pathway.

## Discussion

Incorrect kinetochore-microtubule attachments must be detected and corrected to ensure accurate chromosome segregation. Tension is key to the process because attachments lacking tension are recognized as incorrect and destabilized, while those under tension are stabilized. We previously found that kinetochore-associated Stu2 is required for the intrinsic selectivity of kinetochores for tension-bearing attachments in vitro [9,10]. Here, we identify a *stu2* mutant that disrupts kinetochore-associated Stu2 function, and find that perturbing these activities in cells leads to biorientation defects. Our findings indicate that kinetochore-bound Stu2 plays an important role in error correction and suggest that the intrinsic tension-sensing mechanism, displayed by purified kinetochores in vitro, plays an important role in establishing bioriented attachments in vivo.

### Identification of a *stu2* mutant that perturbs kinetochore function yet maintains normal spindle structure

Stu2’s multiple cellular functions have complicated our ability to examine its kinetochore-specific role since *stu2* mutants disrupt spindle structure [12–14]. Here, we circumvented these challenges by screening for *stu2* mutants that disrupt kinetochore binding using in vitro kinetochore binding assays, which are performed in the absence of microtubules. One of the mutants we identified, *stu2*^*ccΔ*^, appears to have a normal mitotic spindle length in cells, giving us a tool to examine Stu2’s kinetochore function in vivo without secondary defects due to altered spindle structure. The s*tu2*^*ccΔ*^ mutant is defective in kinetochore biorientation, consistent with its importance for intrinsic tension-sensing in vitro. Although this mutant was reported to have reduced microtubule polymerase activity in vitro [18], our data suggests that cells can compensate for this because 1) the mitotic spindle length was normal, and 2) inactivation of the spindle checkpoint allowed the *stu2*^*ccΔ*^ mutant cells to segregate chromosomes, consistent with normal spindle function. In addition, partially restoring the in vitro polymerase activity by adding positively charged resides to the basic linker domain did not improve the *stu2*^*ccΔ*^ biorientation defects in vivo, suggesting that the polymerase activity is not related to the kinetochore function. In the future, this can be tested by identifying a mutant that disrupts kinetochore function yet displays normal in vitro microtubule polymerase activity. Our data identify the *stu2*^*ccΔ*^ allele as the first mutant that supports a normal mitotic spindle structure in cells yet shows defects in kinetochore biorientation.

### Stu2 utilizes multiple domains to achieve kinetochore association

We found that Stu2 utilizes multiple domains to achieve kinetochore binding because the Stu2^ccΔ^, Stu2^TOG1Δ^, and Stu2^C*-*termΔ^ mutants all perturbed kinetochore association, results that are largely consistent with recent work examining Stu2-kinetochore proximity using fluorescent microscopy [22,41]. While the TOG1 domain is not strictly required for Stu2’s ability to bind the kinetochore, we routinely observed decreased Stu2-kinetochore association in its absence. In contrast to [22], however, our data indicate that tubulin binding of the TOG domains is not required for kinetochore localization; a mutant that disrupts TOG domain-tubulin binding did not affect Stu2 binding to kinetochores. The discrepancy is likely because microscopy in yeast cannot discriminate between the kinetochore- and microtubule tip-bound pools, whereas our system analyzes kinetochore localization directly. We also found that the coiled-coil domain is required for kinetochore localization, but this region of the protein does not mediate direct kinetochore binding because it can be replaced with an exogenous homo-dimerization motif. We therefore propose that Stu2’s C-terminal tail domain (residues 855–888) mediates direct kinetochore association, and that two copies of this domain are required to “sandwich” its binding motif on Ndc80c. Consistent with this, prior FRET-based observations also pointed to proximity between the C-terminus of Stu2 and the C-termini of Ndc80 and Nuf2 [42]. However, the C-terminus has additional binding partners that affect microtubule function (Bik1 and Bim1 binding to the C-terminal domain of Stu2; [20,21,32]), precluding analysis of a specific kinetochore function.

### Regulation of Stu2 kinetochore function

Our results strongly indicate that Stu2 has an important role in biorientation; how Stu2’s kinetochore-associated function is regulated, however, remains unanswered. An interesting observation that may shed light on this question is related to the SK-rich region-Ndc80c interaction. We observed that Stu2’s SK-rich region is dispensable for kinetochore binding, yet the majority of crosslinks we observed by XL-MS are between this domain and a region near Ndc80’s conserved hairpin region. Recently, the SK-rich region was found to be involved in an intra-molecular regulation of Stu2, in which tubulin occupancy of the TOG domains regulates the ability of the SK-rich domain to bind to the microtubule lattice [18]. An intriguing idea is that intra-kinetochore tension and/or TOG domain-tubulin occupancy alters the ability of the SK-rich domain to interact with Ndc80’s hairpin domain, thus regulating the combined function of these proteins in microtubule tip binding. Future work examining the interaction between these domains will be important for understanding how Stu2’s kinetochore function is regulated.

It has been proposed that Stu2's function at the kinetochore is solely facilitated by regulation of the microtubule tip [22]. Consistent with this, there is a relatively high turnover of kinetochore-proximal Stu2 observed in cells [22,42]. However, Stu2 binds to Ndc80c in vitro in a stoichiometric manner that does not require microtubules [10], suggesting a kinetochore-bound pool. Additionally, recent data suggest that outer kinetochore stoichiometry is actually quite dynamic in cells [43,44]. One possibility is that Stu2’s kinetochore-association is highly regulated, consistent with turnover rates changing dramatically over different cell cycle stages [42]. Further analysis of Stu2 mutants should help to separate the pools and determine the precise functions for the protein at distinct cellular sites.

### Multiple pathways promote biorientation in cells

Erroneous microtubule attachments must be selectively destabilized by the cell’s error correction machinery until all attachments become correctly bioriented (reviewed in [2]). Error correction requires the activity of the conserved kinase Aurora B, but whether this pathway is solely responsible for correcting erroneous attachments has been less clear. Here, we show that Stu2 also functions at kinetochores in vivo to promote kinetochore biorientation and error correction [9,10]. Reminiscent of the requirements of Aurora B [8], Stu2 is required for the establishment of bioriented attachments but dispensable for their maintenance. We found that the Stu2-dependent mechanism works together with the Aurora B-mediated pathway in the process of error correction, and it will be critical to determine whether they function in parallel and/or temporally to promote biorientation, or whether Aurora B activity directly regulates kinetochore-associated Stu2 function. We propose that the Stu2-dependent intrinsic tension-sensing mechanism constitutes a primordial error correction system used by cells to ensure accurate chromosome segregation prior to evolving the Aurora B-mediated pathway. Given that XMAP215 family members, including human ch-TOG, display a conserved association with the kinetochore’s Ndc80c [10,22,26,27,41,42], it will be important to investigate whether intrinsic tension-selectivity is a wide-spread feature of kinetochore-microtubule interactions, whether mutations that alter ch-TOG regulation [45,46] lead to defects in error correction, and whether these defects correlate with the increased rates of chromosome mis-segregation observed in most cancers.

## Materials and methods

### Strain construction and microbial techniques

#### Yeast strains and plasmids

*Saccharomyces cerevisiae* strains used in this study are described in S1 Table and are derivatives of SBY3 (W303). Standard media and microbial techniques were used [48]. Yeast strains were constructed by standard genetic techniques. Construction of *pCUP1-GFP-LacI* and *ipl1-321* are described in [4], *CEN3*::*lacO*:*TRP1* and *CEN8*::*lacO*:*TRP1* are described in [49], *MTW1-3GFP*, *TUB1-CFP* and *mad3Δ* are described in [37], *DSN1-6His-3Flag* is described in [9], *stu2-3V5-IAA7*, *stu2-3HA-IAA7*, *and SPC24-6His-3Flag* were constructed by PCR-based methods [50] and are described in [10]. Strains containing previously described alleles were also generously provided (*cdc20-IAA17* from Eris Duro and Adèle Marston; *SPC42-CFP* from Sue Jaspersen; *pMET-CDC20* from Frank Uhlmann). *pADH1-TIR1-9Myc* is described in [51]. *pGPD1-TIR1* integration plasmids (pSB2271 for integration at *LEU2*, pSB2273 for integration at *HIS3* or pSB2275 for integration at *TRP1*) as well as a 3V5-IAA7 tagging plasmid (pSB2065) were provided by Leon Chan. Construction of a 3HA-IAA7 tagging plasmid (pSB2229) was described previously in [10]. Construction of a *LEU2* integrating plasmid containing wild-type *pSTU2-STU2-3V5* (pSB2232) is also described in [10]. *STU2* variants were constructed by mutagenizing pSB2232 as described in [52,53], resulting in pSB2254 (*pSTU2-stu2(Δ2–281)-3V5*, i.e. *stu2*^*TOG1Δ*^), pSB2255 (*pSTU2-stu2(Δ2–550)-3V5*, i.e. *stu2*^*TOG1-2Δ*^), pSB2257 (*pSTU2-stu2(Δ282–550*::*GDGAGL*^*linker*^*)-3V5*, i.e. *stu2*^*TOG2Δ*^), pSB2259 (*pSTU2-stu2(Δ551–657*::*GDGAGL*^*linker*^*)-3V5*, i.e. *stu2*^*SK-richΔ*^), pSB2261 (*pSTU2-stu2(Δ658–761*::*GDGAGL*^*linker*^*)-3V5*, i.e. *stu2*^*ccΔ*^), pSB2306 (*pSTU2-stu2(R200A)-3V5*, i.e. *stu2*^*R200A*^), pSB2307 (*pSTU2-stu2(R519A)-3V*5, i.e. *stu2*^*R519A*^), pSB2308 (*pSTU2-stu2(R200A R519A)-3V5*, i.e. *stu2*^*R200A R519A*^), pSB2309 (*pSTU2-stu2(Δ658–761*::*GDGAGL*^*linker*^*-GCN4(249–281)*^*bZIP*^*-GDGAGL*^*linker*^*)-3V5*, i.e. *stu2*^*ccΔ*::*bZIP*^), pSB2357 (*pSTU2-stu2(Δ762–888)-3V5*, i.e. *stu2*^*C-termΔ(762–888)*^), pSB2358 (*pSTU2-stu2(Δ855–888)-3V5*, i.e. *stu2*^*C-termΔ(855–888)*^), pSB3103 (*pSTU2-stu2(2x basic_Δ658–761*::*GDGAGL*^*linker*^*)-3V5*, i.e. *stu2*^*2x basic_ccΔ*^) (“2x basic” as described in [18]). Primers used in the construction of the above plasmids are listed in S2 Table, further details of plasmid construction including plasmid maps available upon request.

#### Auxin inducible degradation

The auxin inducible degron (AID) system was used essentially as described [51]. Briefly, cells expressed C-terminal fusions of the protein of interest to an auxin responsive protein (IAA7 or IAA17) at the endogenous locus. Cells also expressed TIR1, which is required for auxin-induced degradation. 100–500 μM IAA (indole-3-acetic acid dissolved in DMSO; Sigma) was added to media to induce degradation of the AID-tagged protein. Auxin was added for 30 min prior to harvesting cells or as is indicated in figure legends.

#### Spotting assay

For the spotting assay, the desired strains were grown overnight in yeast extract peptone plus 2% glucose (YPD) medium. The following day, cells were diluted to OD_600_ ~1.0 from which a serial 1:5 dilution series was made and spotted on YPD+DMSO or YPD+100–500 μM IAA (indole-3-acetic acid dissolved in DMSO) plates. Plates were incubated at 23–30°C for 2–3 days unless otherwise noted.

#### Cell fixation and imaging conditions

Exponentially growing cultures were treated with 500 μM auxin for 2h, then fixed (see below) and analyzed for Mtw1-3GFP localization and spindle morphology (Spc42-CFP or Tub1-CFP). An aliquot of cells was fixed with 3.7% formaldehyde in 100mM phosphate buffer (pH 6.4) for 5 min. Cells were washed once with 100mM phosphate (pH 6.4), resuspended in 100mM phosphate, 1.2M sorbitol buffer (pH 7.5) and permeabilized with 1% Triton X-100 stained with 1 μg/ml DAPI (4', 6-diamidino-2-phenylindole; Molecular Probes). Cells were imaged using a Nikon E600 microscope with a 60X objective (NA = 1.40), equipped with a Photometrics Cascade 512B digital camera. Five Z-stacks (0.3 micron apart) were acquired and all frames with nuclear signal in focus were maximally projected. NIS Elements software (Nikon) was used for image acquisition and processing.

#### Biorientation assay

In metaphase, sister kinetochores become bioriented and are transiently stretched apart by opposing microtubule pulling forces [23–25], which can be visualized by fluorescently marking the centromere of a single chromosome [39]. Biorientation was examined in metaphase arrested cells as judged by the fluorescently marked centromeres appearing as two distinct foci. Cells were grown in YPD (for *cdc20-AID* containing strains) or media lacking methionine (for *pMET-CDC20* containing strains in Fig 5C). Exponentially growing cells were subsequently arrested in metaphase either by the addition of 500μM IAA for 2h (for *cdc20-AID* containing strains in Fig 5A, whereby biorientation was examined); or the addition of 8mM methionine each hour for 3h (for *pMET-CDC20* containing strains in Fig 5C). The *maintenance* of biorientation was determined in *pMET-CDC20* containing strains by the addition of 500μM IAA (to degrade endogenous Stu2-AID protein) for 2h and monitoring biorientation over time.

#### Cell cycle progression and chromosome segregation assay

Cells were grown in YPD medium. Exponentially growing *MATa* cells also carrying a tandem array of lacO sequences integrated either proximal to *CEN3* or *CEN8* [49] and a LacI-GFP fusion [4,39] were arrested in G1 with 1μg/ml α-factor. When arrest was complete, cells were released into medium lacking α-factor pheromone and containing 500μM IAA at either 23°C or 30°C (semi-permissive temperature). ~75 min after G1 release (for 23°C) or ~65 min after G1 release (for 30°C), 1μg/ml α-factor was added to prevent a second cell division. Samples were taken every 15min after G1 release to determine cell cycle progression (via nuclear morphology of DAPI stained nuclei) and chromosome segregation in anaphase (for Fig 4E at 23°C, chromosome segregation at 120 min post-release is quantified; for Fig 6B at 30°C, chromosome segregation at 75 min post-release is quantified).

### Protein biochemistry

#### Purification of native kinetochore particles, Ndc80 complex and Stu2

Native kinetochore particles, Ndc80c and Stu2 were purified from asynchronously growing *S*. *cerevisiae* cells as described below (unless otherwise noted in the text). For kinetochore particles, an α-Flag immunoprecipitation of Dsn1-6His-3Flag was performed (essentially as described in [9]). To purify the Ndc80c, an α-Flag immunoprecipitation of Spc24-6His-3Flag was performed (protein lysate from SBY14022). To purify Stu2, an α-V5 immunoprecipitation of the Stu2-3V5 variant was performed from strains in which the endogenous Stu2-AID protein was degraded (see below). For each, cells were grown in yeast peptone dextrose rich (YPD) medium. For strains containing Stu2-AID or Spc105-AID, cells were treated with 500 μM auxin 30 min prior to harvesting. Protein lysates were prepared by lysing cells in a Freezer/Mill (SPEX SamplePrep) submerged in liquid nitrogen [54] or mechanically disrupted in the presence of lysis buffer using glass beads and a beadbeater (Biospec Products). Lysed cells were resuspended in buffer H (BH) (25 mM HEPES pH 8.0, 2 mM MgCl_2_, 0.1 mM EDTA, 0.5 mM EGTA, 0.1% NP-40, 15% glycerol with 150 mM KCl for native kinetochores, 750 mM KCl for native Ndc80c or 1 M KCl for native Stu2) containing protease inhibitors (at 20 μg mL^-1^ final concentration for each of leupeptin, pepstatin A, chymostatin and 200 μM phenylmethylsulfonyl fluoride) and phosphatase inhibitors (0.1 mM Na-orthovanadate, 0.2 μM microcystin, 2 mM β-glycerophosphate, 1 mM Na pyrophosphate,5 mM NaF) followed by ultracentrifugation at 98,500 g for 90 min at 4°C. Lysates prepared using the beadbeater were instead centrifuged at 16,100 g for 30 min at 4°C. Dynabeads conjugated with α-Flag or α-V5 antibodies were incubated with extract for 3 h with constant rotation, followed by three washes with BH containing protease inhibitors, phosphatase inhibitors, 2 mM dithiothreitol (DTT) and either 150 mM KCl (kinetochores) or 1 M KCl (Ndc80c and Stu2). Beads were further washed twice with BH containing 150 mM KCl and protease inhibitors. Associated proteins were eluted from the beads by gentle agitation of beads in elution buffer (0.5 mg ml^−1^ 3Flag peptide or 0.5 mg ml^−1^ 3V5 peptide in BH with 150 mM KCl and protease inhibitors) for 30 min at room temperature. To eliminate the co-purification of the Spc105 complex with the native Ndc80c, we immunoprecipitated Spc24-6His-3Flag from cells carrying an *spc105-AID* allele that were treated with 500 μM auxin for 30 min.

#### Immunoblot analysis

For immunoblot analysis, cell lysates were prepared as described above (Protein biochemistry section) or by pulverizing cells with glass beads in sodium dodecyl sulfate (SDS) buffer using a bead-beater (Biospec Products). Standard procedures for sodium dodecyl sulfate-polyacrylamide gel electrophoresis (SDS-PAGE) and immunoblotting were followed as described in [55,56]. A nitrocellulose membrane (Bio-Rad) was used to transfer proteins from polyacrylamide gels. Commercial antibodies used for immunoblotting were as follows: α-Flag, M2 (Sigma-Aldrich) 1:3,000; α-V5 (Invitrogen) 1:5,000. Antibodies to Ctf19, and Ndc80 were kind gifts from Arshad Desai and were used at: α-Ctf19, (OD10) 1:1,000; and α-Ndc80, (OD4) 1:10,000. The secondary antibodies *used* were a sheep anti-mouse antibody conjugated to horseradish peroxidase (HRP) (GE Biosciences) at a 1:10,000 dilution or a donkey anti-rabbit antibody conjugated to HRP (GE Biosciences) at a 1:10,000 dilution. Antibodies were detected using the SuperSignal West Dura Chemiluminescent Substrate (Thermo Scientific).

#### In vitro binding assays

To examine the binding of Stu2 to purified kinetochores, Stu2-3V5 and native kinetochores were purified from exponentially growing yeast cells as described above (Protein biochemistry section). Prior to eluting purified kinetochores (with approximately 75–100 ng of Dsn1-Flag bound) from α-Flag dynabeads, beads were incubated with 15 μl (30–90 ng) of purified Stu2-3V5 for 30 min at room temperature with gentile agitation. Beads were then washed twice with BH containing 150 mM KCl and protease inhibitors. Associated proteins were eluted from the beads by gentle agitation of beads in elution buffer (0.5 mg ml^−1^ 3Flag peptide in BH with 150 mM KCl and protease inhibitors) for 30 min at room temperature.

#### Recombinant protein expression and purification

The *S*. *cerevisiae* Ndc80c was expressed in *E*. *coli* using polycistronic vectors, and purified as previously described [34,57,58]. Stu2 constructs were made in pHAT vector containing N-terminal 6His tag, C-terminal eGFP-tag followed by a *Strep*-tag II (the original vector was a gift from Dr. Gary Brouhard). Expression was induced in *E*. *coli* using Arctic Express Cells and purified as in [18].

#### Chemical cross-linking and mass spectrometry analysis (XL-MS)

Cross-linking reactions, mass spectrometry and data analysis were carried out as described previously [59]. A protein mixture containing 3.3 μg Stu2 plus 3 μg Ndc80c in a final volume of 44 μL BRB80 (80 mM PIPES, 1mM EGTA, 1 mM MgCl_2_, 10 μM taxol) was incubated for 15 minutes at room temperature. Cross-linking was initiated by adding 3.75 μL of 145 mM EDC plus 1.88 μL of 145 mM sulfo-NHS dissolved in BRB80. Cross-linking was allowed to proceed for 30 minutes at room temperature before quenching by addition of 5 μL 1M ammonium bicarbonate plus 0.5 μL 2-mercaptoethanol. Samples were reduced with 10 mM dithiothreitol (DTT) for 30 mins at 37°C followed by alkylation with 15 mM iodoacetamide (IAA) for 30 mins at room temperature. Tryptic digestion was performed at 37°C for 4 hours with shaking at a substrate to trypsin ratio of 18:1. After digestion, samples were acidified with 5 M HCl and stored at -80°C until analysis. Mass spectrometry was performed on a Q-Exactive HF (Thermo Fisher Scientific) in data dependent mode by loading 0.4 μg of sample onto a fused-silica capillary tip column (75-μm i.d.) packed with 30 cm of Reprosil-Pur C18-AQ (3-μm bead diameter, Dr. Maisch). Peptides were eluted from the column at 0.25 μl/min using a 120 min acetonitrile gradient. Spectra were converted into mzML using msconvert from ProteoWizard [60]. Proteins present in the sample were identified using Comet [61]. Cross-linked peptides were identified within those proteins using Kojak version 1.4.3 [62] available at (http://www.kojak-ms.org). Percolator version 2.08 [63] was used to assign a statistically meaningful q value to each peptide spectrum match (PSM) through analysis of the target and decoy PSM distributions. The target databases consisted of all proteins identified in the sample. The decoy database consisted of the corresponding set of reversed protein sequences. Data presented here were filtered to show hits to the target proteins that had a Percolator assigned peptide level q value ≤ 0.05. The complete, unfiltered list of all PSMs and their Percolator assigned q values, are available on the ProXL web application [64] at: https://proxl.yeastrc.org/proxl/viewProject.do?project_id=52 along with the raw MS spectra and search parameters used.

### Optical trap assays

#### Bead preparation for optical trap assays

Optical trap-based bead motility assays were performed as in [9,10,65]. Streptavidin-coated 0.44-μm polystyrene beads (Spherotech) were functionalized with biotinylated anti-penta-His antibody (Qiagen) and decorated with purified native Ndc80c (via Spc24-6His-3Flag). Bead decoration was performed in a total volume of 20 μl incubation buffer (BRB80 containing 1 mg ml^-1^ κ-casein). 10 nM of purified native Ndc80c was incubated with 6 pM beads for 1 h at 4°C, unbound protein was removed by pelleting the beads (16,000 g for 10 min at 4°C), washing with ~200 μl of incubation buffer, pelleting beads again (16,000 g for 10 min at 4°C) and resuspending in original volume. For the addition of purified Stu2 to Ndc80c, Ndc80c-decorated beads were prepared as above and purified Stu2-3V5 was added to the microtubule growth buffer (see below) to a final concentration of 2 nM as in [10].

#### Rupture force measurements

Dynamic microtubule extensions were grown from coverslip-anchored GMPCPP-stabilized microtubule seeds in a microtubule growth buffer consisting of BRB80, 1mM GTP, 250 μg ml^−1^ glucose oxidase, 25 mM glucose, 30 μg ml^−1^ catalase, 1mM DTT, 1.4–1.5 mg ml^−1^ purified bovine brain tubulin, and blocking protein (1 mg ml^−1^ κ-casein). Assays were performed at 23°C. Rupture force experiments were performed as in [9,10,47,54]. Briefly, an optical trap was used to apply a force of ∼2 pN in the direction of microtubule assembly. Once beads were observed to track with microtubule growth for a distance of ~100–300 nm (to ensure end-on attachment), the applied force was increased at a constant rate of 0.25 pN s^-1^ until bead detachment. Records of bead position over time were collected and analyzed using custom software (Labview and Igor Pro, respectively) and used to determine the rupture force, which was marked as the maximum force sustained by the attachment during each event. All the individual rupture force values and calculated mean rupture strengths are provided in S3 Table.

## Supporting information

S1 Fig Stu2’s kinetochore association depends on homo-dimerization and its extreme C-terminus. A)Protein lysates were prepared from exponentially growing *stu2-AID* cultures expressing various *STU2-3V5* alleles from an ectopic locus treated with auxin 30 min prior to harvesting (*STU2*^*WT*^, SBY13557; *stu2*^*TOG1Δ*^, SBY13563; *stu2*^*TOG2Δ*^, SBY13569; *stu2*^*TOG1-2Δ*^, SBY13575; *stu2*^*SK-richΔ*^, SBY13581; *stu2*^*ccΔ*^, SBY13587). Stu2-3V5 was purified by α-V5 IP, followed by washes in buffer containing 1.0 M KCl (BH 1.0) then V5 peptide elution. Eluate was run on an SDS-PAGE gel and analyzed by silver stain. Background bands were previously determined by mass spectrometry to be the highly homologous heat shock proteins Ssa1, Ssa2 (70 kDa), and Ssb1, Ssb2 (66 kDa), which are common co-purifying proteins in IPs from yeast lysates [5], and were isolated in all purifications. B) As in (A) expressing different *STU2-3V5* alleles (*stu2*^*R200A*^, SBY13923; *stu2*^*R519A*^, SBY13929; *stu2*^*R200A R519A*^, SBY13933; *stu2*^*ccΔ*::*bZIP*^, SBY13939; *stu2*^*C-termΔ(762–888)*^, SBY14267; *stu2*^*C-termΔ(855–888)*^, SBY14273). C) Protein lysates were prepared from exponentially growing cultures containing Dsn1-6His-3Flag Stu2-AID (SBY13772) treated with 500 μM auxin for 30 min prior to harvesting cells. Kinetochore particles were immobilized by α-Flag IP. Immobilized kinetochore-beads were incubated with 30 ng of Stu2-3V5 variants (purified as in A) for 30 min at room temperature, washed and eluted with Flag peptide. Kinetochore-bound proteins were analyzed by immunoblotting with α-Flag, α-V5, α-Ndc80 and α-Ctf19 antibodies. Note: Stu2^SK-rich*Δ*^ and Dsn1-6His-3Flag co-migrate on an SDS-PAGE gel. For technical reasons (that are not entirely clear), detection of Dsn1-6His-3Flag was affected by first probing for Stu2^SK-rich*Δ*^, however similar Dsn1-6His-3Flag levels were observed for all samples when these sample eluates were run on an SDS-PAGE gel and analyzed by silver stain. D) As in (C) using Stu2-3V5 variants (purified as in B). E) Exponentially growing *stu2-AID* cultures expressing an ectopic copy of Stu2 (*STU2*^*WT*^, SBY13901; *stu2*^*TOG1Δ*^, SBY13904; *stu2*^*R200A*^, SBY13919; *stu2*^*TOG2Δ*^, SBY13907; *stu2*^*R519A*^, SBY13925; *stu2*^*TOG1-2Δ*^, SBY13910; *stu2*^*R200A R519A*^, SBY13930; *stu2*^*SK-richΔ*^, SBY13913; *stu2*^*ccΔ*^, SBY13916; *stu2*^*ccΔ*::*bZIP*^, SBY13935 or *stu2*^*C-termΔ(855–888)*^, SBY14269) or without an ectopic allele (no covering allele, SBY13772) that also contained Dsn1-6His-3Flag were treated with auxin 30 min prior to harvesting. Protein lysates were subsequently prepared and kinetochore particles were purified by α-Flag immunoprecipitation (IP) and analyzed by immunoblotting. F) Protein lysates were prepared from exponentially growing cultures containing Spc24-6His-3Flag Spc105-AID (SBY14022) treated with 500 μM auxin for 30 min prior to harvesting cells. Ndc80c was immobilized by α-Flag IP. Immobilized Ndc80c-beads were incubated with 30 ng of Stu2-3V5 variants (purified as in A) for 30 min at room temperature, washed and eluted with Flag peptide. Ndc80c-bound proteins were analyzed by immunoblotting with α-Flag, α-V5, and α-Ndc80 antibodies. G) Summary table of kinetochore binding and viability data from Fig 1, S1 Fig and S2 Fig.
(TIF)

S2 Fig Phenotype of various *stu2* mutants. A) Schematic of Stu2’s domain architecture. B) Wild-type (SBY3), *stu2-AID* (no covering allele, SBY13772) and *stu2-AID* cells expressing various *STU2-3V5* alleles from an ectopic locus (*STU2*^*WT*^, SBY13901; *stu2*^*SK-richΔ*^, SBY13913; *stu2*^*ccΔ*^, SBY13916; *stu2*^*ccΔ*::*bZIP*^, SBY13935; *stu2*^*TOG1Δ*^, SBY13904; *stu2*^*R200A*^, SBY13919; *stu2*^*TOG2Δ*^, SBY13907; *stu2*^*R519A*^, SBY13925; *stu2*^*TOG1-2Δ*^, SBY13910; *stu2*^*R200A R519A*^, SBY13930) were serially diluted (5-fold) and spotted on YPD or 5 μg/ml benomyl plates containing either DMSO or 100 μM auxin. C) Wild-type (SBY3), *stu2-AID* (no covering allele, SBY13772) and *stu2-AID* cells expressing various *STU2-3V5* alleles from an ectopic locus [*STU2*^*WT*^, SBY13901; *stu2*^*C-termΔ(762–888)*^, SBY14263; *stu2*^*C-termΔ(855–888)*^, SBY14269) were serially diluted (5-fold) and spotted on YPD or 5 μg/ml benomyl plates containing either DMSO or 100 μM auxin.
(TIF)

S3 Fig Cell cycle progression of kinetochore-binding deficient Stu2 mutant. A) Kinetochore distribution (distance between bi-lobed kinetochore clusters) and spindle length (spindle pole-to-pole distance) was measured for cells described in (Figs 2B & 4C). n = 80–105 cells; now plotted as an X-Y scatter plot to compare distances between bi-lobed kinetochore clusters as a function of spindle length. Dashed lines are a best fit linear regression for each data set. B-F) Cell cycle progression from Fig 4D. Quantification of the number of mononucleate and binucleate cells for each strain. Shown is a representative experiment.
(TIF)

S4 Fig Dimerization deficient mutant with increased polymerase activity phenocopies *stu2^ccΔ^* and cellular Stu2 function is required to maintain a normal bipolar spindle and bi-lobed kinetochore distribution. A) Wild-type (SBY3), *stu2-AID* (no covering allele, SBY13772) and *stu2-AID* cells expressing various *STU2-3V5* alleles from an ectopic locus (*STU2*^*WT*^, SBY13901; *stu2*^*ccΔ*^, SBY13916; *stu2*^*2x basic_ccΔ*^, SBY19025) were serially diluted (5-fold) and spotted on plates containing either DMSO or 500 μM auxin. B) Exponentially growing Stu2-AID cultures with an ectopic copy of *STU2* (*STU2*^*WT*^, SBY13901; *stu2*^*ccΔ*^, SBY13916; or *stu2*^*2x basic_ccΔ*^, SBY19025) that also contained Dsn1-6His-3Flag were treated with auxin 30 min prior to harvesting. Protein lysates were subsequently prepared and kinetochore particles were purified by α-Flag immunoprecipitation (IP) and analyzed by immunoblotting. C) Exponentially growing *stu2-AID pMET-CDC20* cells that contained a fluorescently labeled *CEN3* and an ectopically expressed *STU2* allele (*STU2*^*WT*^, SBY18370; *stu2*^*ccΔ*^, SBY18371; or *stu2*^*2x basic_ccΔ*^, SBY19058) were arrested in metaphase by the addition of methionine for 3 h. Concurrent with methionine addition, auxin was added to degrade the Stu2-AID protein and the percentage of cells that display two distinct GFP foci (i.e. bioriented *CEN3*) was quantified. Error bars represent SD of three independent experiments; n = 150–200 cells for each time point. Note: We used the Stu2-AID system to examine the effect of depleting all cellular Stu2 on spindle structure after the cells had already formed a mitotic spindle. For these experiments, we arrested cells in metaphase by depleting Cdc20 (now using a methionine repressible *pMET-CDC20* allele) and found that the subsequent degradation of the Stu2-AID protein led to a significant decrease in both spindle length and collapse of the bi-lobed kinetochore clusters to a mono-lobed focus (S4D–S4F Fig). A recent study used an “anchor away” system to address this same question [22]. However, we repeated this experiment because that study only observed a 70% mis-localization of Stu2 by fluorescence microscopy and no alteration in mitotic spindle length, suggesting incomplete depletion of Stu2 from the nucleus. This previous study [22] proposed a model for Stu2’s role in the tension-dependent stabilization of microtubule attachments that we previously reported [9,10]. Essentially, their model relies on Stu2 being a microtubule destabilizing factor that, by inducing curled protofilaments, provides a “flared” tip for the kinetochore to bind. In this model attachments to assembling tips are weak due to the absence of this “flared” tip structure, and thus, kinetochores require Stu2 for long lived attachments at higher forces by inducing a compatible binding structure. While this is an intriguing model, it appears inconsistent with our previous observations that attachments of purified kinetochores to assembling microtubule tips are stronger even in the absence of Stu2 [9,10,47]. Additionally, this model only explains how Stu2 would promote longer lived attachments at high force, and does not appear compatible with the observation that kinetochore associated Stu2 also appears to destabilize low force-bearing attachments. Finally, while there is in vitro evidence that Stu2 can act as a microtubule destabilizing factor in the absence of free tubulin [31], there is limited evidence that Stu2 acts as a destabilizing factor in cells. D) Exponentially growing cells carrying *pMET-CDC20 MTW1-3GFP TUB1-CFP* and either the wild-type *STU2* (SBY17105) or *stu2-AID* allele (SBY17106) at the endogenous *STU2* locus were arrested in metaphase by depleting Cdc20 (by the addition of methionine to the media). Once cells were arrested in metaphase, auxin was added to induce degradation of the Stu2-AID protein, and the cells were subsequently analyzed for kinetochore distribution and spindle morphology (by examining Mtw1-3GFP and Tub1-CFP, respectively). Representative images for each are shown for pre-auxin and 60 min post-auxin addition. Kinetochore distribution was determined to be bi- or mono-lobed. E) Kinetochore distribution (distance between bi-lobed kinetochore clusters) was measured for cells described in (A). n = 46–63 cells; p values were determined using a two-tailed unpaired t test (n.s. = not significant). F) Spindle length (length of Tub1-CFP) was measured for cells described in (A). n = 38–41 cells; p value was determined using a two-tailed unpaired t test.
(TIF)

S5 Fig *stu2^ccΔ^* mutant displays synthetic phenotype with an Aurora B mutant. Wild-type (SBY3), *stu2-AID* (no covering allele, SBY13772) and *stu2-AID* cells expressing various *STU2-3V5* alleles from an ectopic locus (*STU2*^*WT*^, SBY13903; *stu2*^*ccΔ*^, SBY13918) or also containing an *ipl1-321* allele (*stu2*^*ccΔ*^ *ipl1-321*, SBY17100) or *ipl1-321* alone (SBY630) were serially diluted (5-fold) and spotted on YPD or 5 μg/ml benomyl plates containing either DMSO or 500 μM auxin and incubated at 23°C (permissive) or 30°C (semi-permissive).
(TIF)

S1 Table Strains used in this study. (DOCX)

S2 Table Plasmids and Primers used in this study. (XLSX)

S3 Table Summary of optical trap-based bead motility assays, related to Fig 3. (XLSX)
