# Supplementary figures and images for: Kinetochore-associated Stu2 promotes chromosome biorientation in vivo
Source: PLoS Genet. 2019 Oct 4;15(10):e1008423. doi: 10.1371/journal.pgen.1008423 (PMC6795502; doi:10.1371/journal.pgen.1008423)

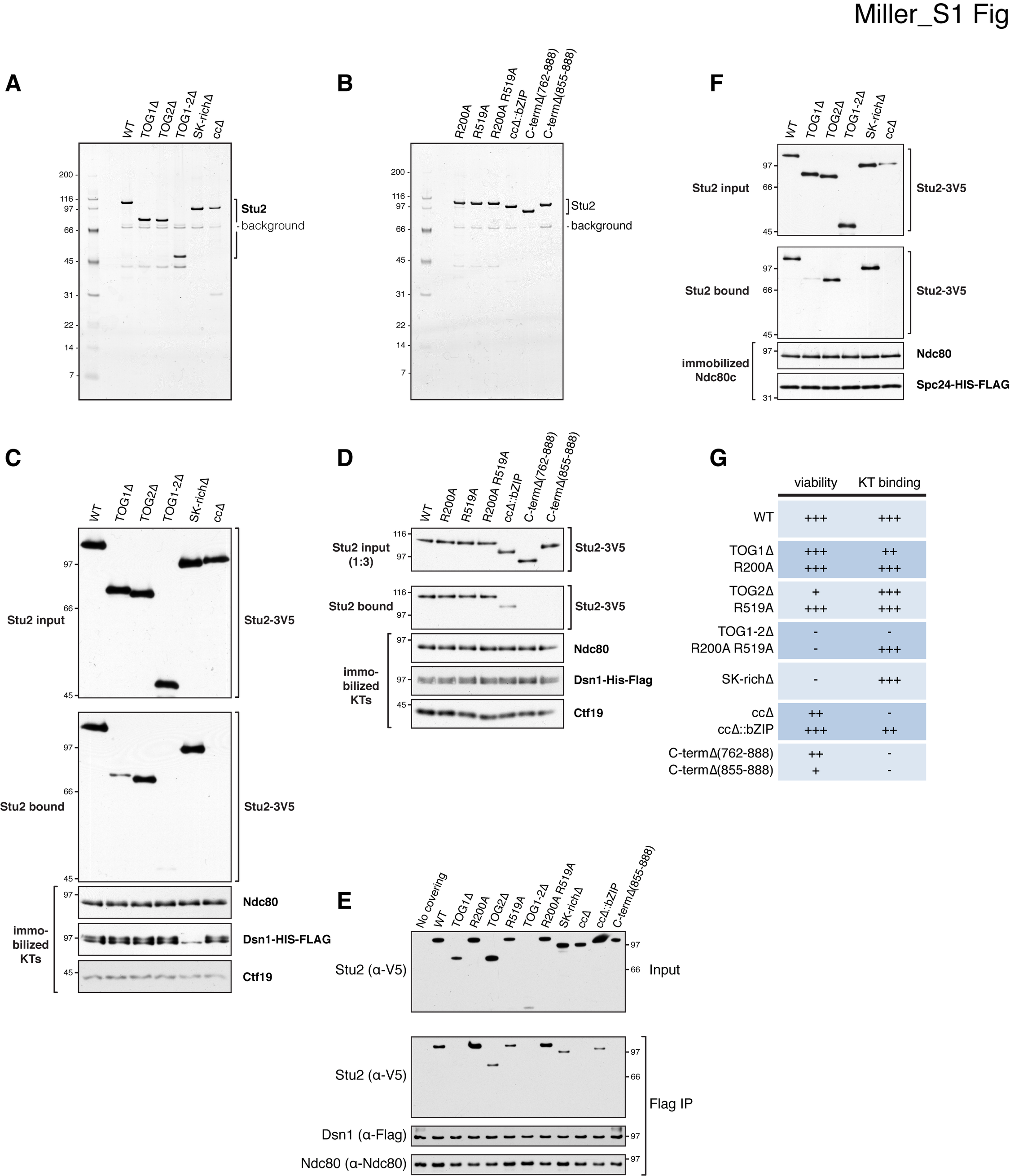

Supplement: S1 Fig — A)Protein lysates were prepared from exponentially growing stu2-AID cultures expressing various STU2-3V5 alleles from an ectopic locus treated with auxin 30 min prior to harvesting (STU2WT, SBY13557; stu2TOG1Δ, SBY13563; stu2TOG2Δ, SBY13569; stu2TOG1-2Δ, SBY13575; stu2SK-richΔ, SBY13581; stu2ccΔ, SBY13587). Stu2-3V5 was purified by α-V5 IP, followed by washes in buffer containing 1.0 M KCl (BH 1.0) then V5 peptide elution. Eluate was run on an SDS-PAGE gel and analyzed by silver stain. Background bands were previously determined by mass spectrometry to be the highly homologous heat shock proteins Ssa1, Ssa2 (70 kDa), and Ssb1, Ssb2 (66 kDa), which are common co-purifying proteins in IPs from yeast lysates [5], and were isolated in all purifications. B) As in (A) expressing different STU2-3V5 alleles (stu2R200A, SBY13923; stu2R519A, SBY13929; stu2R200A R519A, SBY13933; stu2ccΔ::bZIP, SBY13939; stu2C-termΔ(762–888), SBY14267; stu2C-termΔ(855–888), SBY14273). C) Protein lysates were prepared from exponentially growing cultures containing Dsn1-6His-3Flag Stu2-AID (SBY13772) treated with 500 μM auxin for 30 min prior to harvesting cells. Kinetochore particles were immobilized by α-Flag IP. Immobilized kinetochore-beads were incubated with 30 ng of Stu2-3V5 variants (purified as in A) for 30 min at room temperature, washed and eluted with Flag peptide. Kinetochore-bound proteins were analyzed by immunoblotting with α-Flag, α-V5, α-Ndc80 and α-Ctf19 antibodies. Note: Stu2SK-richΔ and Dsn1-6His-3Flag co-migrate on an SDS-PAGE gel. For technical reasons (that are not entirely clear), detection of Dsn1-6His-3Flag was affected by first probing for Stu2SK-richΔ, however similar Dsn1-6His-3Flag levels were observed for all samples when these sample eluates were run on an SDS-PAGE gel and analyzed by silver stain. D) As in (C) using Stu2-3V5 variants (purified as in B). E) Exponentially growing stu2-AID cultures expressing an ectopic copy of Stu2 (STU2WT, SBY13901; stu2TOG1Δ, SBY [file pgen.1008423.s001.tif]

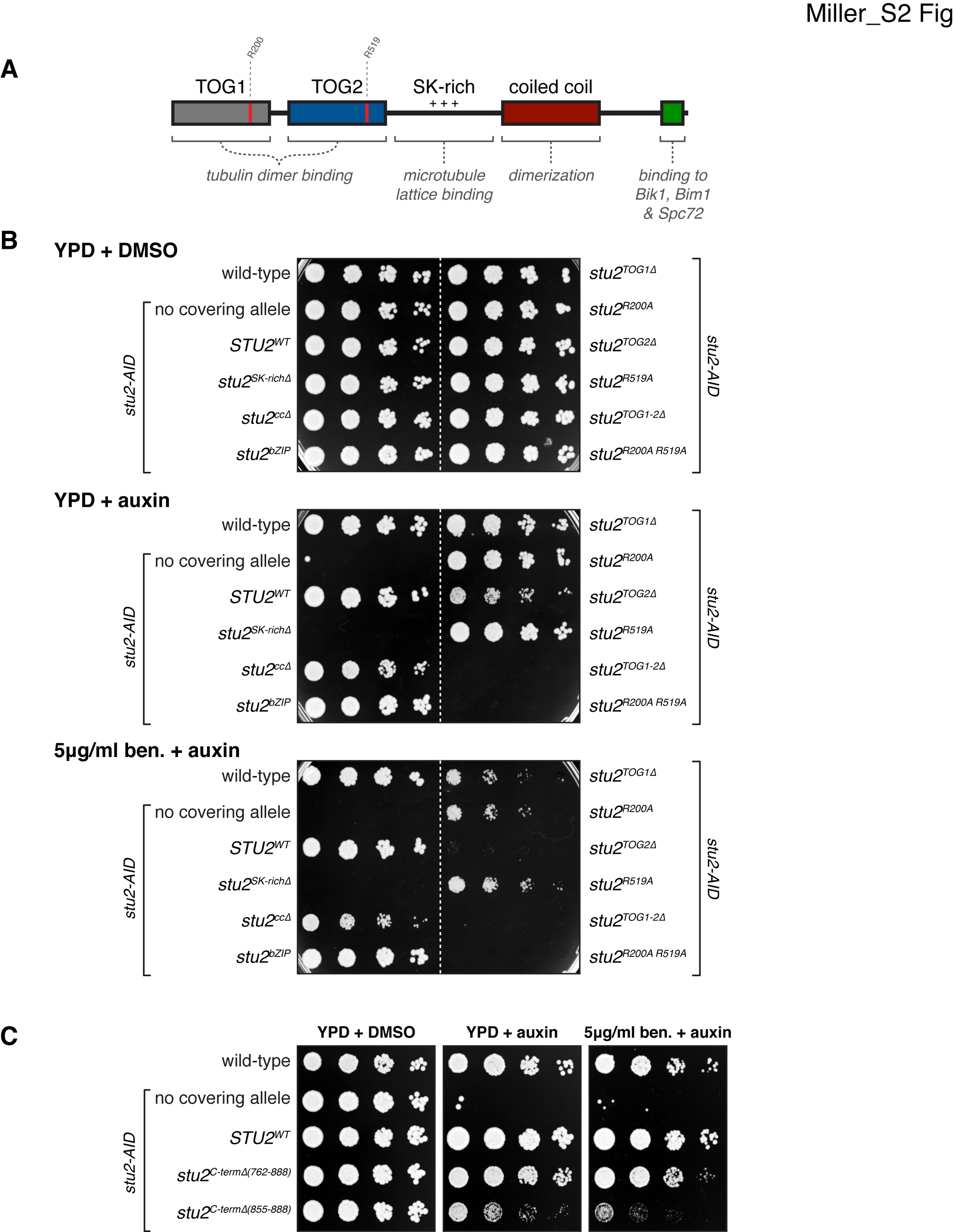

Supplement: S2 Fig — A) Schematic of Stu2’s domain architecture. B) Wild-type (SBY3), stu2-AID (no covering allele, SBY13772) and stu2-AID cells expressing various STU2-3V5 alleles from an ectopic locus (STU2WT, SBY13901; stu2SK-richΔ, SBY13913; stu2ccΔ, SBY13916; stu2ccΔ::bZIP, SBY13935; stu2TOG1Δ, SBY13904; stu2R200A, SBY13919; stu2TOG2Δ, SBY13907; stu2R519A, SBY13925; stu2TOG1-2Δ, SBY13910; stu2R200A R519A, SBY13930) were serially diluted (5-fold) and spotted on YPD or 5 μg/ml benomyl plates containing either DMSO or 100 μM auxin. C) Wild-type (SBY3), stu2-AID (no covering allele, SBY13772) and stu2-AID cells expressing various STU2-3V5 alleles from an ectopic locus [STU2WT, SBY13901; stu2C-termΔ(762–888), SBY14263; stu2C-termΔ(855–888), SBY14269) were serially diluted (5-fold) and spotted on YPD or 5 μg/ml benomyl plates containing either DMSO or 100 μM auxin. (TIF) [file pgen.1008423.s002.tif]

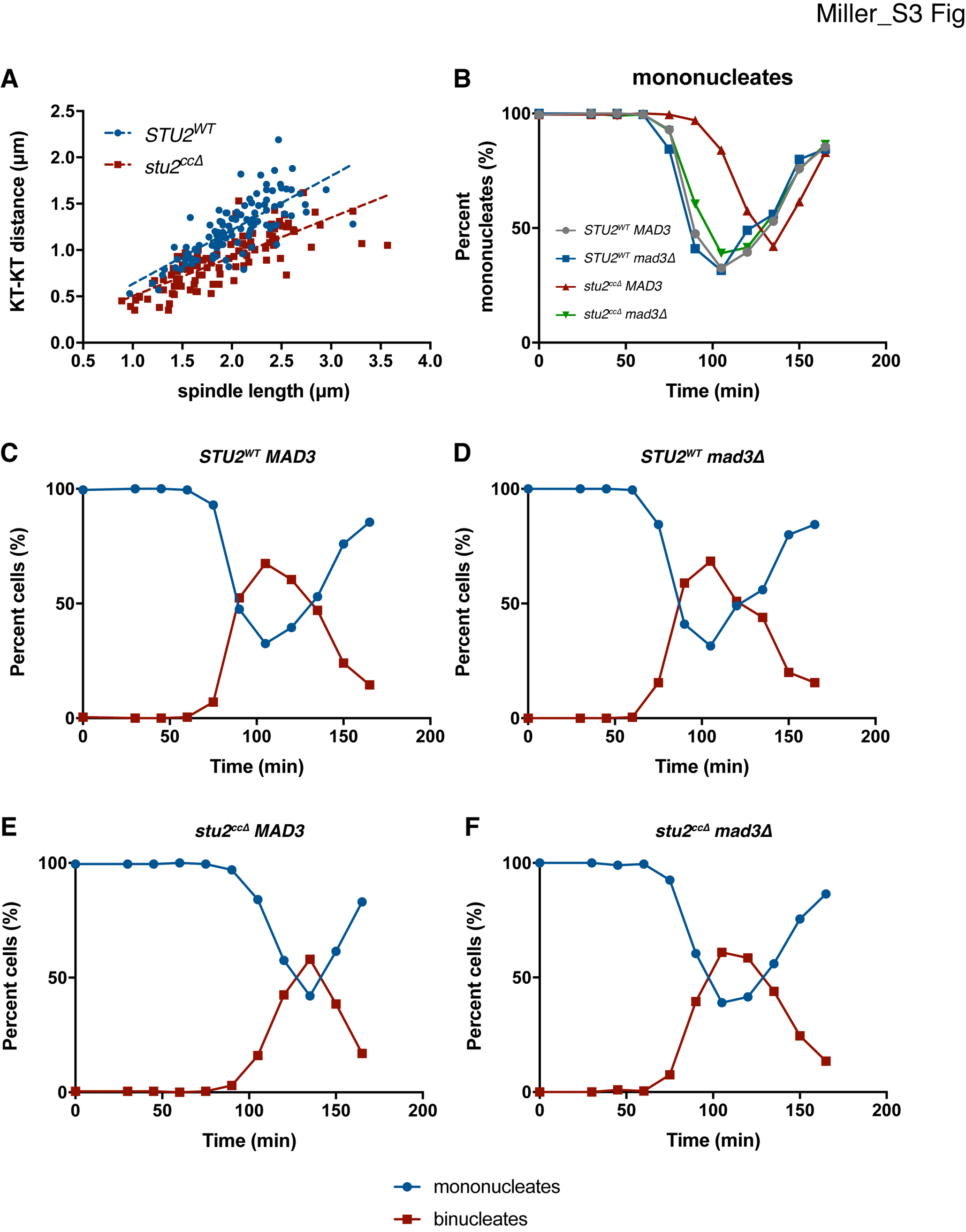

Supplement: S3 Fig — A) Kinetochore distribution (distance between bi-lobed kinetochore clusters) and spindle length (spindle pole-to-pole distance) was measured for cells described in (Figs 2B & 4C). n = 80–105 cells; now plotted as an X-Y scatter plot to compare distances between bi-lobed kinetochore clusters as a function of spindle length. Dashed lines are a best fit linear regression for each data set. B-F) Cell cycle progression from Fig 4D. Quantification of the number of mononucleate and binucleate cells for each strain. Shown is a representative experiment. (TIF) [file pgen.1008423.s003.tif]

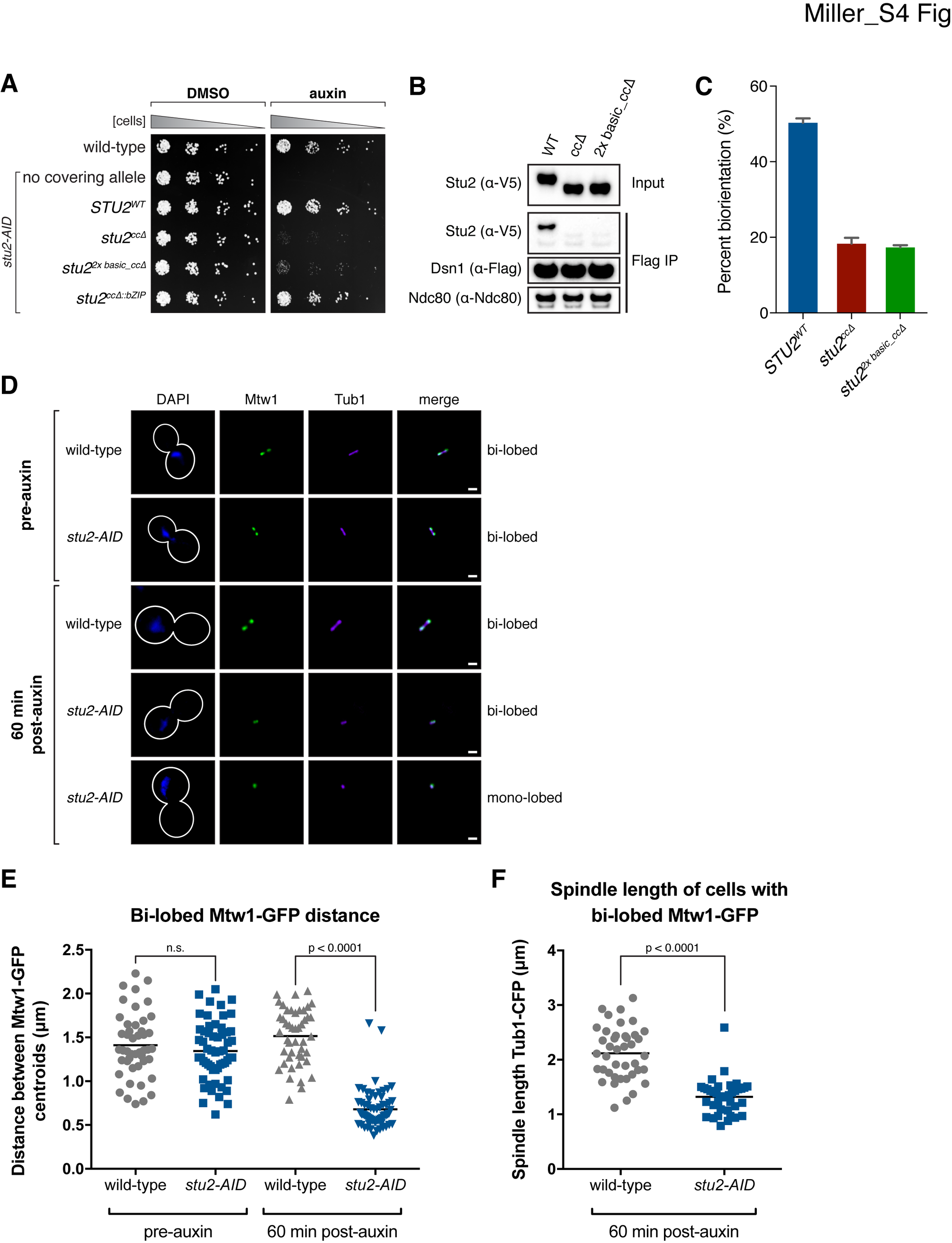

Supplement: S4 Fig — A) Wild-type (SBY3), stu2-AID (no covering allele, SBY13772) and stu2-AID cells expressing various STU2-3V5 alleles from an ectopic locus (STU2WT, SBY13901; stu2ccΔ, SBY13916; stu22x basic_ccΔ, SBY19025) were serially diluted (5-fold) and spotted on plates containing either DMSO or 500 μM auxin. B) Exponentially growing Stu2-AID cultures with an ectopic copy of STU2 (STU2WT, SBY13901; stu2ccΔ, SBY13916; or stu22x basic_ccΔ, SBY19025) that also contained Dsn1-6His-3Flag were treated with auxin 30 min prior to harvesting. Protein lysates were subsequently prepared and kinetochore particles were purified by α-Flag immunoprecipitation (IP) and analyzed by immunoblotting. C) Exponentially growing stu2-AID pMET-CDC20 cells that contained a fluorescently labeled CEN3 and an ectopically expressed STU2 allele (STU2WT, SBY18370; stu2ccΔ, SBY18371; or stu22x basic_ccΔ, SBY19058) were arrested in metaphase by the addition of methionine for 3 h. Concurrent with methionine addition, auxin was added to degrade the Stu2-AID protein and the percentage of cells that display two distinct GFP foci (i.e. bioriented CEN3) was quantified. Error bars represent SD of three independent experiments; n = 150–200 cells for each time point. Note: We used the Stu2-AID system to examine the effect of depleting all cellular Stu2 on spindle structure after the cells had already formed a mitotic spindle. For these experiments, we arrested cells in metaphase by depleting Cdc20 (now using a methionine repressible pMET-CDC20 allele) and found that the subsequent degradation of the Stu2-AID protein led to a significant decrease in both spindle length and collapse of the bi-lobed kinetochore clusters to a mono-lobed focus (S4D–S4F Fig). A recent study used an “anchor away” system to address this same question [22]. However, we repeated this experiment because that study only observed a 70% mis-localization of Stu2 by fluorescence microscopy and no alteration in mitotic spindle length, suggesting incomplet [file pgen.1008423.s004.tif]

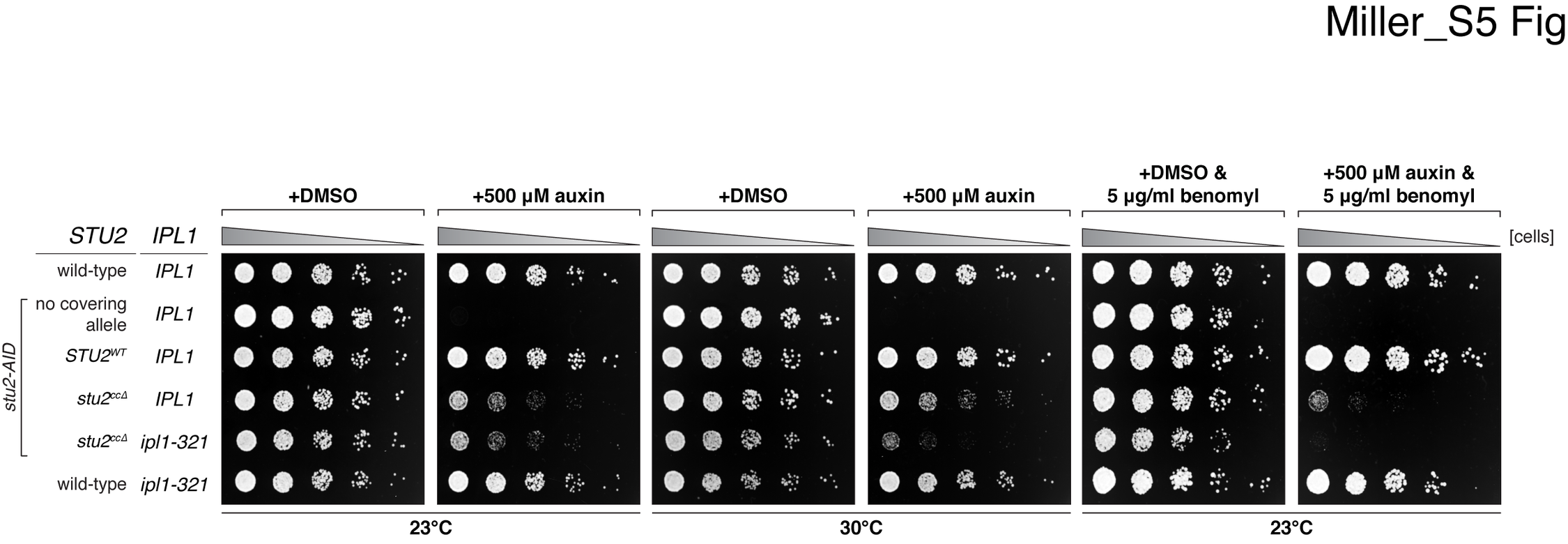

Supplement: S5 Fig — Wild-type (SBY3), stu2-AID (no covering allele, SBY13772) and stu2-AID cells expressing various STU2-3V5 alleles from an ectopic locus (STU2WT, SBY13903; stu2ccΔ, SBY13918) or also containing an ipl1-321 allele (stu2ccΔ ipl1-321, SBY17100) or ipl1-321 alone (SBY630) were serially diluted (5-fold) and spotted on YPD or 5 μg/ml benomyl plates containing either DMSO or 500 μM auxin and incubated at 23°C (permissive) or 30°C (semi-permissive). (TIF) [file pgen.1008423.s005.tif]
