# Supplementary material for: Kinetochore-associated Stu2 promotes chromosome biorientation in vivo
Source: PLoS Genet. 2019 Oct 4;15(10):e1008423. doi: 10.1371/journal.pgen.1008423 (PMC6795502; doi:10.1371/journal.pgen.1008423)
Supplement: S1 Table — (DOCX) [file pgen.1008423.s006.docx]

**S1 Table.** Strains used in this study.

All strains are derivatives of SBY3 (W303)

**Strain Relevant Genotype**

| SBY3 (W303) | *MAT*a *ura3-1 leu2-3,112 his3-11 trp1-1 can1-100 ade2-1 bar1-1* |
| --- | --- |
| SBY630 | *MAT*a *ipl1-321* |
| SBY13557 | *MAT*a *STU2-3HA-IAA7:KanMX his3::pADH1-TIR1-9Myc:HIS3 leu2::pSTU2-STU2-3V5:LEU2* |
| SBY13563 | *MAT*a *STU2-3HA-IAA7:KanMX his3::pADH1-TIR1-9Myc:HIS3 leu2::pSTU2-stu2(∆2-281)-3V5:LEU2* |
| SBY13569 | *MAT*a *STU2-3HA-IAA7:KanMX his3::pADH1-TIR1-9Myc:HIS3 leu2::pSTU2-stu2(∆282-550::GDGAGL^linker^)-3V5:LEU2* |
| SBY13575 | *MAT*a *STU2-3HA-IAA7:KanMX his3::pADH1-TIR1-9Myc:HIS3 leu2::pSTU2-stu2(∆2-550)-3V5:LEU2* |
| SBY13581 | *MAT*a *STU2-3HA-IAA7:KanMX his3::pADH1-TIR1-9Myc:HIS3 leu2::pSTU2-stu2(∆551-657::GDGAGL^linker^)-3V5:LEU2* |
| SBY13587 | *MAT*a *STU2-3HA-IAA7:KanMX his3::pADH1-TIR1-9Myc:HIS3 leu2::pSTU2-stu2(∆658-761::GDGAGL^linker^)-3V5:LEU2* |
| SBY13772 | *MAT*a *STU2-3HA-IAA7:KanMX DSN1-6His-3Flag:URA3 his3::pGPD1-TIR1:HIS3* |
| SBY13901 | *MAT*a *STU2-3HA-IAA7:KanMX DSN1-6His-3Flag:URA3 his3::pGPD1-TIR1:HIS3 leu2::pSTU2-STU2-3V5:LEU2* |
| SBY13903 | *MAT*a *STU2-3HA-IAA7:KanMX his3::pGPD1-TIR1:HIS3 leu2::pSTU2-STU2-3V5:LEU2* |
| SBY13904 | *MAT*a *STU2-3HA-IAA7:KanMX DSN1-6His-3Flag:URA3 his3::pGPD1-TIR1:HIS3 leu2::pSTU2-stu2(∆2-281)-3V5:LEU2* |
| SBY13907 | *MAT*a *STU2-3HA-IAA7:KanMX DSN1-6His-3Flag:URA3 his3::pGPD1-TIR1:HIS3 leu2::pSTU2-stu2(∆282-550::GDGAGL^linker^)-3V5:LEU2* |
| SBY13910 | *MAT*a *STU2-3HA-IAA7:KanMX DSN1-6His-3Flag:URA3 his3::pGPD1-TIR1:HIS3 leu2::pSTU2-stu2(∆2-550)-3V5:LEU2* |
| SBY13913 | *MAT*a *STU2-3HA-IAA7:KanMX DSN1-6His-3Flag:URA3 his3::pGPD1-TIR1:HIS3 leu2::pSTU2-stu2(∆551-657::GDGAGL^linker^)-3V5:LEU2* |
| SBY13916 | *MAT*a *STU2-3HA-IAA7:KanMX DSN1-6His-3Flag:URA3 his3::pGPD1-TIR1:HIS3 leu2::pSTU2-stu2(∆658-761::GDGAGL^linker^)-3V5:LEU2* |
| SBY13918 | *MAT*a *STU2-3HA-IAA7:KanMX his3::pGPD1-TIR1:HIS3 leu2::pSTU2-stu2(∆658-761::GDGAGL^linker^)-3V5:LEU2* |
| SBY13919 | *MAT*a *STU2-3HA-IAA7:KanMX DSN1-6His-3Flag:URA3 his3::pGPD1-TIR1:HIS3 leu2::pSTU2-stu2(R200A)-3V5:LEU2* |
| SBY13923 | *MAT*a *STU2-3HA-IAA7:KanMX his3::pGPD1-TIR1:HIS3 leu2::pSTU2-stu2(R200A)-3V5:LEU2* |
| SBY13925 | *MAT*a *STU2-3HA-IAA7:KanMX DSN1-6His-3Flag:URA3 his3::pGPD1-TIR1:HIS3 leu2::pSTU2-stu2(R519A)-3V5:LEU2* |
| SBY13929 | *MAT*a *STU2-3HA-IAA7:KanMX his3::pGPD1-TIR1:HIS3 leu2::pSTU2-stu2(R519A)-3V5:LEU2* |
| SBY13930 | *MAT*a *STU2-3HA-IAA7:KanMX DSN1-6His-3Flag:URA3 his3::pGPD1-TIR1:HIS3 leu2::pSTU2-stu2(R200A R519A)-3V5:LEU2* |
| SBY13933 | *MAT*a *STU2-3HA-IAA7:KanMX his3::pGPD1-TIR1:HIS3 leu2::pSTU2-stu2(R200A R519A)-3V5:LEU2* |
| SBY13935 | *MAT*a *STU2-3HA-IAA7:KanMX DSN1-6His-3Flag:URA3 his3::pGPD1-TIR1:HIS3 leu2::pSTU2-stu2(∆658-761::GDGAGL^linker^-GCN4(249-281)^bZIP^-GDGAGL^linker^)-3V5:LEU2* |
| SBY13939 | *MAT*a *STU2-3HA-IAA7:KanMX his3::pGPD1-TIR1:HIS3 leu2::pSTU2-stu2(∆658-761::GDGAGL^linker^-GCN4(249-281)^bZIP^-GDGAGL^linker^)-3V5:LEU2* |
| SBY14022 | *MAT*a *SPC24-6His-3Flag:URA3 SPC105-3HA-IAA7:KanMX trp1::pGPD1-TIR1:TRP1* |
| SBY14263 | *MAT*a *STU2-3HA-IAA7:KanMX DSN1-6His-3Flag:URA3 his3::pGPD1-TIR1:HIS3 leu2::pSTU2-stu2(∆762-888)-3V5:LEU2* |
| SBY14267 | *MAT*a *STU2-3HA-IAA7:KanMX his3::pGPD1-TIR1:HIS3 leu2::pSTU2-stu2(∆762-888)-3V5:LEU2* |
| SBY14269 | *MAT*a *STU2-3HA-IAA7:KanMX DSN1-6His-3Flag:URA3 his3::pGPD1-TIR1:HIS3 leu2::pSTU2-stu2(∆855-888)-3V5:LEU2* |
| SBY14273 | *MAT*a *STU2-3HA-IAA7:KanMX his3::pGPD1-TIR1:HIS3 leu2::pSTU2-stu2(∆855-888)-3V5:LEU2* |
| SBY17100 | *MAT*a *STU2-3HA-IAA7:KanMX his3::pGPD1-TIR1:HIS3 leu2::pSTU2-stu2(∆658-761::GDGAGL^linker^)-3V5:LEU2 ipl1-321* |
| SBY17105 | *MAT*a *pMET-CDC20:TRP1 MTW1-3GFP:HIS3 ura3::TUB1-CFP:URA3 leu2::pGPD1-TIR1:LEU2* |
| SBY17106 | *MAT*a *pMET-CDC20:TRP1 MTW1-3GFP:HIS3 ura3::TUB1-CFP:URA3 leu2::pGPD1-TIR1:LEU2 STU2-3V5-IAA7:KanMX* |
| SBY17367 | *MAT*a *CDC20-IAA17:KanMX STU2-3V5-IAA7:KanMX MTW1-3GFP:HIS3 SPC42-CFP:HygMX trp1::pGPD1-TIR1:TRP1* |
| SBY17369 | *MAT*a *CDC20-IAA17:KanMX STU2-3V5-IAA7:KanMX MTW1-3GFP:HIS3 SPC42-CFP:HygMX trp1::pGPD1-TIR1:TRP1 leu2::pSTU2-STU2-3V5:LEU2* |
| SBY17371 | *MAT*a *CDC20-IAA17:KanMX STU2-3V5-IAA7:KanMX MTW1-3GFP:HIS3 SPC42-CFP:HygMX trp1::pGPD1-TIR1:TRP1 leu2::pSTU2-stu2(∆658-761::GDGAGL^linker^)-3V5:LEU2* |
| SBY17527 | *MAT*a *STU2-3V5-IAA7:KanMX trp1::pGPD1-TIR1:TRP1 ura3::TUB1-CFP:URA3 his3::pCUP1-GFP-LacI:HIS3 CEN8::lacO:TRP1 leu2::pSTU2-STU2-3V5:LEU2* |
| SBY17560 | *MAT*a *STU2-3V5-IAA7:KanMX trp1::pGPD1-TIR1:TRP1 ura3::TUB1-CFP:URA3 his3::pCUP1-GFP-LacI:HIS3 CEN8::lacO:TRP1 leu2::pSTU2-stu2(∆658-761::GDGAGL^linker^)-3V5:LEU2* |
| SBY17668 | *MAT*a *STU2-3V5-IAA7:KanMX trp1::pGPD1-TIR1:TRP1 ura3::TUB1-CFP:URA3 his3::pCUP1-GFP-LacI:HIS3 CEN8::lacO:TRP1 leu2::pSTU2-STU2-3V5:LEU2 mad3∆::NatMX* |
| SBY17669 | *MAT*a *STU2-3V5-IAA7:KanMX trp1::pGPD1-TIR1:TRP1 ura3::TUB1-CFP:URA3 his3::pCUP1-GFP-LacI:HIS3 CEN8::lacO:TRP1 leu2::pSTU2-stu2(∆658-761::GDGAGL^linker^)-3V5:LEU2 mad3∆::NatMX* |
| SBY17708 | *MAT*a *STU2-3V5-IAA7:KanMX CDC20-IAA17:KanMX trp1::pGPD1-TIR1:TRP1 ura3::TUB1-CFP:URA3 his3::pCUP1-GFP-LacI:HIS3 CEN8::lacO:TRP1* |
| SBY17748 | *MAT*a *STU2-3V5-IAA7:KanMX CDC20-IAA17:KanMX trp1::pGPD1-TIR1:TRP1 ura3::TUB1-CFP:URA3 his3::pCUP1-GFP-LacI:HIS3 CEN8::lacO:TRP1 leu2::pSTU2-STU2-3V5:LEU2* |
| SBY17750 | *MAT*a *STU2-3V5-IAA7:KanMX CDC20-IAA17:KanMX trp1::pGPD1-TIR1:TRP1 ura3::TUB1-CFP:URA3 his3::pCUP1-GFP-LacI:HIS3 CEN8::lacO:TRP1 leu2::pSTU2-stu2(∆658-761::GDGAGL^linker^)-3V5:LEU2* |
| SBY18242 | *MAT*a *STU2-3V5-IAA7:KanMX trp1::pGPD1-TIR1:TRP1 ura3::TUB1-CFP:URA3 his3::pCUP1-GFP-LacI:HIS3 CEN3::lacO:TRP1 mad3∆::HygMX leu2::pSTU2-STU2-3V5:LEU2* |
| SBY18244 | *MAT*a *STU2-3V5-IAA7:KanMX trp1::pGPD1-TIR1:TRP1 ura3::TUB1-CFP:URA3 his3::pCUP1-GFP-LacI:HIS3 CEN3::lacO:TRP1 mad3∆::HygMX leu2::pSTU2-STU2-3V5:LEU2 ipl1-321* |
| SBY18246 | *MAT*a *STU2-3V5-IAA7:KanMX trp1::pGPD1-TIR1:TRP1 ura3::TUB1-CFP:URA3 his3::pCUP1-GFP-LacI:HIS3 CEN3::lacO:TRP1 mad3∆::HygMX leu2::pSTU2-stu2(∆658-761::GDGAGL^linker^)-3V5:LEU2* |
| SBY18248 | *MAT*a *STU2-3V5-IAA7:KanMX trp1::pGPD1-TIR1:TRP1 ura3::TUB1-CFP:URA3 his3::pCUP1-GFP-LacI:HIS3 CEN3::lacO:TRP1 mad3∆::HygMX leu2::pSTU2-stu2(∆658-761::GDGAGL^linker^)-3V5:LEU2 ipl1-321* |
| SBY18359 | *MAT*a *pMET-CDC20:TRP1 STU2-3V5-IAA7:KanMX trp1::pGPD1-TIR1:TRP1 his3::pCUP1-GFP-LacI:HIS3 CEN3::lacO:TRP1* |
| SBY18370 | *MAT*a *pMET-CDC20:TRP1 STU2-3V5-IAA7:KanMX trp1::pGPD1-TIR1:TRP1 his3::pCUP1-GFP-LacI:HIS3 CEN3::lacO:TRP1 leu2::pSTU2-STU2-3V5:LEU2* |
| SBY18371 | *MAT*a *pMET-CDC20:TRP1 STU2-3V5-IAA7:KanMX trp1::pGPD1-TIR1:TRP1 his3::pCUP1-GFP-LacI:HIS3 CEN3::lacO:TRP1 leu2::pSTU2-stu2(∆658-761::GDGAGL^linker^)-3V5:LEU2* |
| SBY19025 | *MAT*a *STU2-3HA-IAA7:KanMX DSN1-6His-3Flag:URA3 his3::pGPD1-TIR1:HIS3 leu2::pSTU2-stu2(2x basic_∆658-761::GDGAGL^linker^)-3V5:LEU2* |
| SBY19058 | *MAT***a** *pMET-CDC20:TRP1 STU2-3V5-IAA7:KanMX trp1::pGPD1-TIR1:TRP1 his3::pCUP1-GFP-LacI:HIS3 CEN3::lacO:TRP1 leu2::pSTU2-stu2(2x basic_∆658-761::GDGAGL^linker^)-3V5:LEU2* |
